# PEGylated IL-10 Activates Kupffer Cells to Control Hypercholesterolemia

**DOI:** 10.1371/journal.pone.0156229

**Published:** 2016-06-14

**Authors:** Ivan H. Chan, Dennis Van Hoof, Marina Abramova, Melissa Bilardello, Elliot Mar, Brett Jorgensen, Scott McCauley, Harminder Bal, Martin Oft, Peter Van Vlasselaer, John B. Mumm

**Affiliations:** ARMO BioSciences, Inc., 575 Chesapeake Drive, Redwood City, CA, 94063, United States of America; University of Maryland, UNITED STATES

## Abstract

Interleukin-10 (IL-10) is a multifunctional cytokine that exerts potent context specific immunostimulatory and immunosuppressive effects. We have investigated the mechanism by which PEGylated rIL-10 regulates plasma cholesterol in mice and humans. In agreement with previous work on rIL-10, we report that PEGylated rIL-10 harnesses the myeloid immune system to control total plasma cholesterol levels. We have discovered that PEG-rMuIL-10’s dramatic lowering of plasma cholesterol is dependent on phagocytotic cells. In particular, PEG-rHuIL-10 enhances cholesterol uptake by Kupffer cells. In addition, removal of phagocytotic cells dramatically increases plasma cholesterol levels, suggesting for the first time that immunological cells are implicitly involved in regulating total cholesterol levels. These data suggest that treatment with PEG-rIL-10 potentiates endogenous cholesterol regulating cell populations not currently targeted by standard of care therapeutics. Furthermore, we show that IL-10’s increase of Kupffer cell cholesterol phagocytosis is concomitant with decreases in liver cholesterol and triglycerides. This leads to the reversal of early periportal liver fibrosis and facilitates the restoration of liver health. These data recommend PEG-rIL-10 for evaluation in the treatment of fatty liver disease and preventing its progression to non-alcoholic steatohepatitis. In direct confirmation of our in vivo findings in the treatment of hypercholesterolemic mice with PEG-rMuIL-10, we report that treatment of hypercholesterolemic cancer patients with PEG-rHuIL-10 lowers total plasma cholesterol by up to 50%. Taken together these data suggest that PEG-rIL-10’s cholesterol regulating biology is consistent between mice and humans.

## Introduction

Interleukin-10 (IL-10) is a pluripotent immuno-regulatory protein initially characterized as an immunosuppressive cytokine. This moniker is predominantly derived from IL-10’s inhibition of Toll-like receptor agonist stimulation of pro-inflammatory cytokine secretion from monocytes [1–4]. Pegylated-rMuIL-10 is a 5kDa N-terminally PEGylated form of IL-10 that has been reported to exert potent CD8+ T cell and IFNƔ dependent anti-tumor immunity [5]. PEGylated rHuIL-10, is currently undergoing immunoncology Phase 1b trials [6]. The IL-10 receptor is expressed by hematopoietic cells [7], epithelial cells [8], fibroblasts [9], hepatic stellate cells [10], and hepatocytes [11]. IL-10 exposure has been previously shown to decrease serum cholesterol and reduce atherosclerotic plaques in Ldlr-/- and ApoE-/- mice [12–14]. Schering Plough safely treated approximately 2500 autoimmune patients with non-PEGylated IL-10 and reported the lowering of total cholesterol by up to 30% [15]. To date however, the underlying mechanism of how IL-10 lowers plasma cholesterol in both mice and humans is unknown. IL-10 has been shown to increase the uptake and efflux of acetylated and oxidized low density lipoprotein (LDL) cholesterol from atherosclerotic lesions via the induction of scavenger receptors in myeloid cells and cell lines [16–18]. This process is termed Reverse Cholesterol Transport (RCT) [19, 20]. Macrophages contribute to RCT through the uptake of both acetylated and oxidized cholesterol [21, 22]. However, treatment with PEG-rIL-10 lowers total cholesterol, not just the acetylated and oxidized forms. Therefore, while IL-10 enhances peripheral macrophage uptake and efflux of modified cholesterol, this biology alone is not sufficient to explain IL-10’s regulation of total cholesterol levels. We observed that Schering-Plough investigators noted a general increase in CD14+ peripheral monocytes in their investigators brochure. Interestingly, increases in peripheral monocytes are associated with low total cholesterol levels in patients suffering from Gaucher disease [23]. Contrary to current theories with regard to the ratio of low density (LDL) to high density (HDL) serum lipoproteins, low HDL in these patients also does not imperil their cardiovascular health [24]. In addition, patients suffering from acute myelogenous leukemia are often hypocholesterolemic [25, 26], with evidence of enhanced cholesterol catabolism [27]. Lastly, the potent mediator of myeloid function GM-CSF [28], is reported to reduce serum cholesterol [29]. We have PEGylated recombinant murine IL-10, (PEG-rMuIL-10), and human IL-10 (AM0010), to improve their pharmacokinetic profiles for the purposes of treating cancer patients. We observed similar total cholesterol reductions in our patients as reported by Schering Plough, and have investigated how PEGylated recombinant IL-10 (PEG-rIL-10) regulates total plasma cholesterol. We provide evidence herein that administration of PEG-rIL-10 enhances liver resident Kupffer cell phagocytosis which reduces total cholesterol levels in hypercholesterolemic mice and humans, thereby engaging endogenous cholesterol regulating cell populations not currently targeted by standard of care therapeutics.

## Results

### PEG-rIL-10 treatment is safe and reduces plasma cholesterol levels

**Fig 1:**
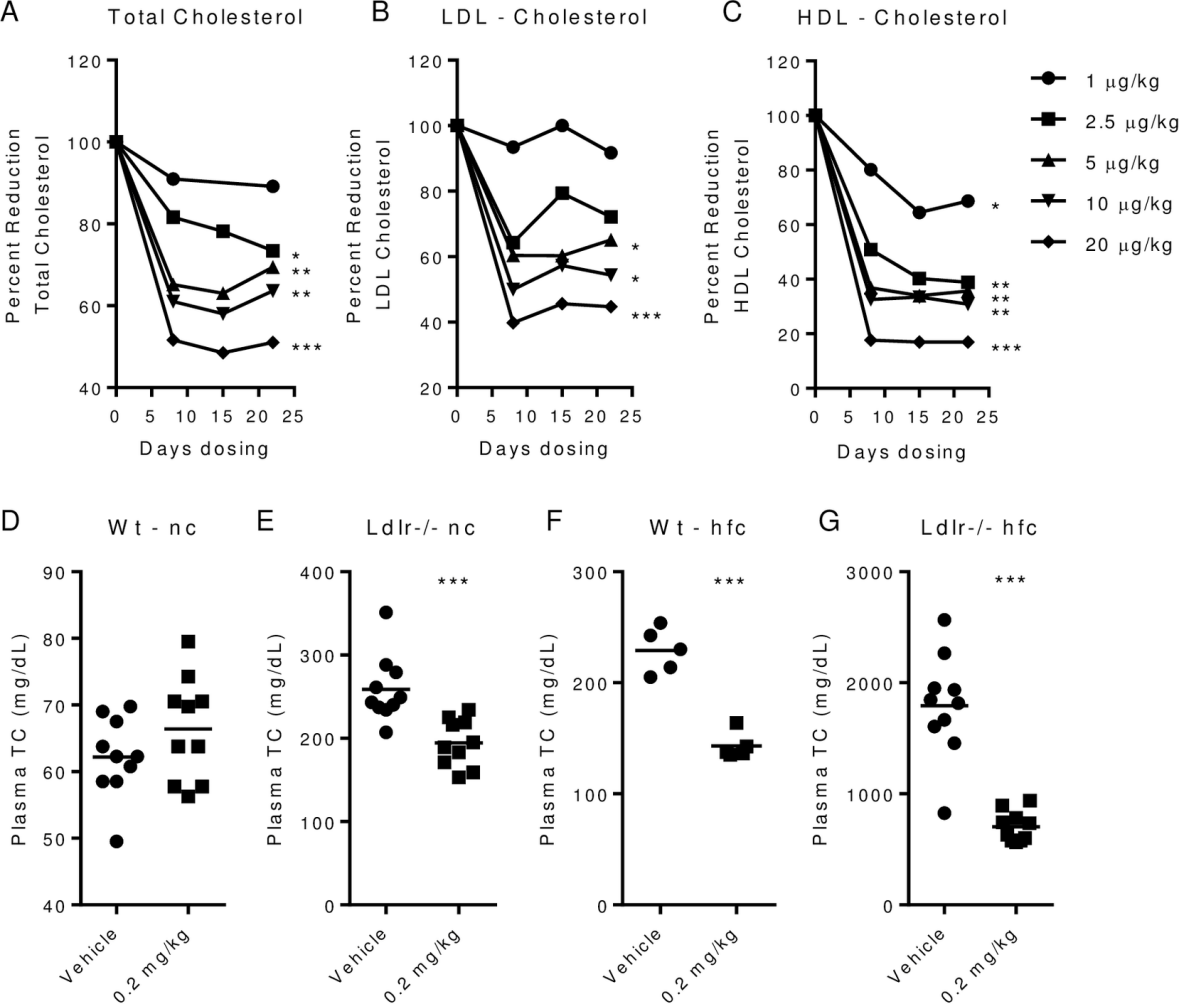
PEG-rIL-10 treatment is safe and reduces plasma cholesterol levels. (A), total plasma cholesterol of cancer patients that received daily subcutaneous doses of 1, 2.5, 5, 10 or 20 μg/kg AM0010 for 22 days. (B), LDL levels of patients in (A). (C), HDL levels of patients in (A). (D-G), 5–10 wt and Ldlr-/- mice were either fed normal chow (nc) or high fat chow (hfc) for two weeks prior to dosing. All mice were dosed with vehicle or 0.2 mg/kg PEG-rMuIL-10 for 1–2 weeks. (D), total plasma cholesterol after dosing of wt mice fed nc. (E), total plasma cholesterol after dosing of Ldlr-/- mice fed nc. (F), total plasma cholesterol after dosing in wt mice fed hfc. (G), total plasma cholesterol after dosing of Ldlr-/- mice fed hfc. (A-C) statistics were assessed by ANOVA multiple comparisons where the control was 100% and where *p<0.05, **p<0.01, ***p<0.001. (D-G) statistics assessed by Students t-test.

**Fig 2:**
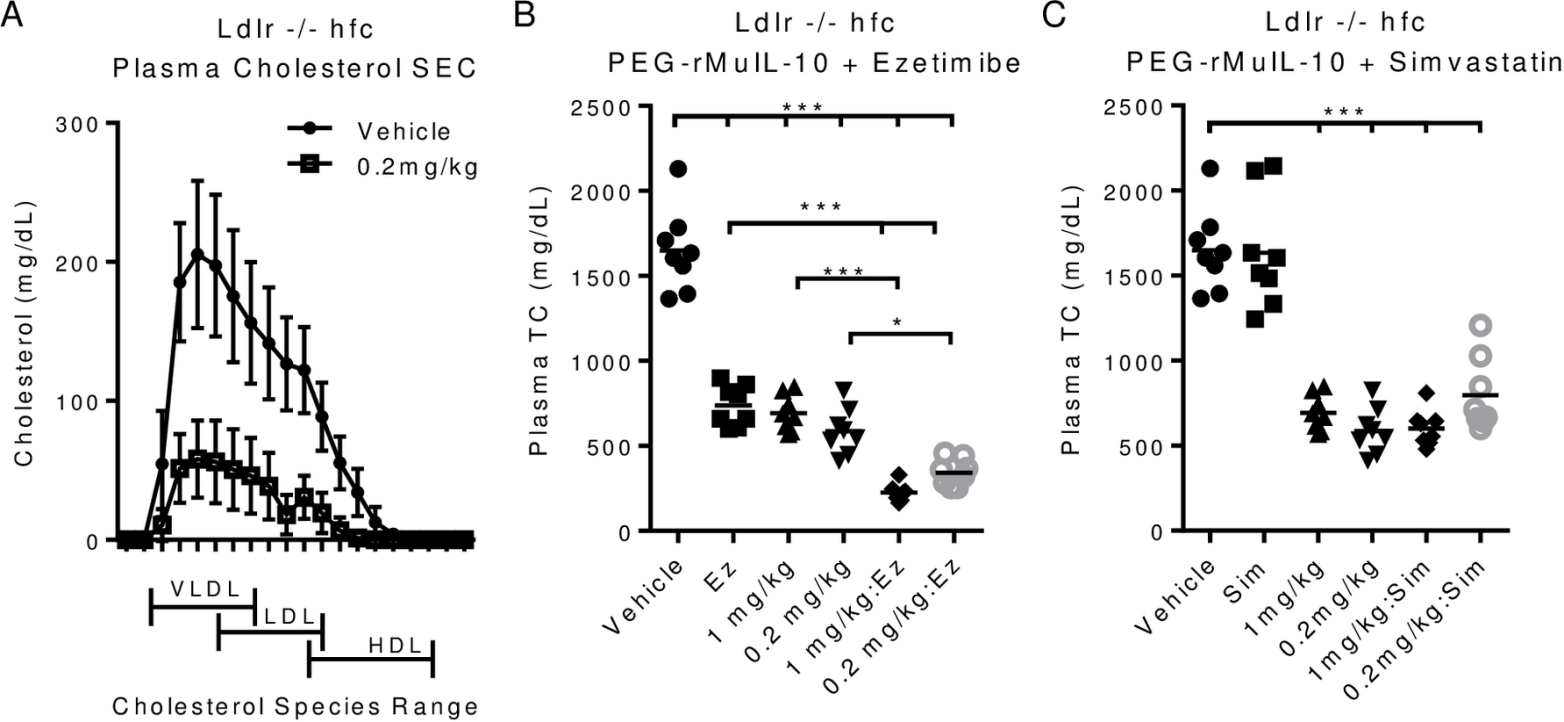
PEG-rMuIL-10 treatment reduces total plasma cholesterol and combines with Ezetimibe. (A), size exclusion chromatographic analysis of plasma cholesterol from vehicle, 0.2 or 1 mg/kg treated Ldlr-/- mice on hfc, treated for 2 weeks. (B), total plasma cholesterol of Ldlr-/- mice treated with 0.2 or 1 mg/kg PEG-rMuIL-10 with or without 10 mg/kg Ezetimibe. (C), total plasma cholesterol of Ldlr-/- mice treated with 0.2 or 1 mg/kg PEG-rMuIL-10 with or without 75 mg/kg Simvastatin. (B-C) statistics assessed by ANOVA multiple comparisons where *p<0.05, **p<0.01, ***p<0.001.

We investigated the effect of AM0010 and PEG-rMuIL-10 treatment on plasma cholesterol in cancer patients, wt and Ldlr-/- mice (Fig 1). Cancer patients exhibit decreases of total, LDL and HDL plasma cholesterol levels of approximately 50% when dosed with up to 20 μg/kg s.c. qd AM0010. (Fig 1A–1C). These data are similar to those reported by Schering Plough, (S1 Table). PEG-rMuIL-10 treated wild type (wt) mice on normal chow exhibit no decrease in total plasma cholesterol (Fig 1D), whereas treatment of moderately hypercholesterolemic low density lipid receptor (Ldlr) -/- mice on normal chow (Fig 1E) and wt mice on high fat chow (Fig 1F) lowers cholesterol by 22–25%. Ldlr-/- mice on high fat chow with plasma cholesterol levels of approximately 1800 mg/dL exhibit a decrease of nearly 50% after two weeks of dosing (Fig 1G). Longer dosing of Ldlr-/- mice leads to total plasma cholesterol drops up to 70% (data not shown). In addition, unlike current therapies such as statins and Ezetimibe, treatment with PEG-rMuIL-10 reduces very low density lipoprotein, (VLDL), LDL and HDL (Fig 2A). Schering Plough reported that recombinant IL-10 treatment was generally well tolerated [30, 31] with chronic treatment lasting up to 1 year (S1 Table). Serum chemistries from Crohn’s patients exhibited few changes when dose subcutaneously daily, for 28 days up to 20 μg/kg (S2 Table) [32–34]. Mice responded similarly to PEG-rMuIL-10 treatment except for a slight increase in murine aspartate aminotransferase (ALT) values, (S3 Table). We have currently treated 266 cancer patients, the longest up to 17 months. The cancer patient dose escalation adverse event profile of 33 patients [6] is consistent with Schering Plough’s reports. Specifically, these data indicate that chronic treatment with AM0010 is safe and well tolerated.

### PEG-rMuIL-10 treatment reduces all plasma cholesterol and combines with Ezetimibe

Given the broad reduction of plasma cholesterol, we investigated whether PEG-rMuIL-10 treatment of high fat fed Ldlr-/- mice exhibited reductions of all plasma lipids, or only a subset of plasma lipids. Treatment with PEG-rMuIL-10 causes the reduction of all forms of plasma lipids (Fig 2A). Given this substantial reduction in all forms of plasma cholesterol we addressed whether PEG-rMuIL-10 was affecting cholesterol uptake by the intestine or the liver. Expression analysis did not reveal any substantial changes to cholesterol uptake or trafficking genes in the jejunum or ileum (S1 and S2 Figs). In support of these data, PEG-rMuIL-10 treatment combines to reduce cholesterol with the uptake blocker Ezetimibe (Fig 2B), but not the hepatic cholesterol synthesis blocker, Simvastatin (Fig 2C). Under the conditions tested, unlike Ezetimibe, Simvastatin did not reduce plasma cholesterol, nor did it combine with PEG-rMuIL-10. These data suggested to us that PEG-rMuIL-10 may not alter the uptake or efflux of dietary cholesterol from the intestinal track. Therefore, given these data and previous investigators reported observations that IL-10 could directly affect cholesterol synthesis biology in human hepatocellular carcinoma cells in vitro, we investigated PEG-rMuIL-10’s effect on liver biology. Previous reports suggested IL-10 regulates the Mevalonate pathway. Expression analysis of the hepatic mevalonate pathway suggests that PEG-rMuIL-10 may moderately inhibit this pathway (S3–S6 Figs).

### PEG-rMuIL-10 regulates cholesterol independent of the scavenger receptors, Msr1 and Marco

**Fig 3:**
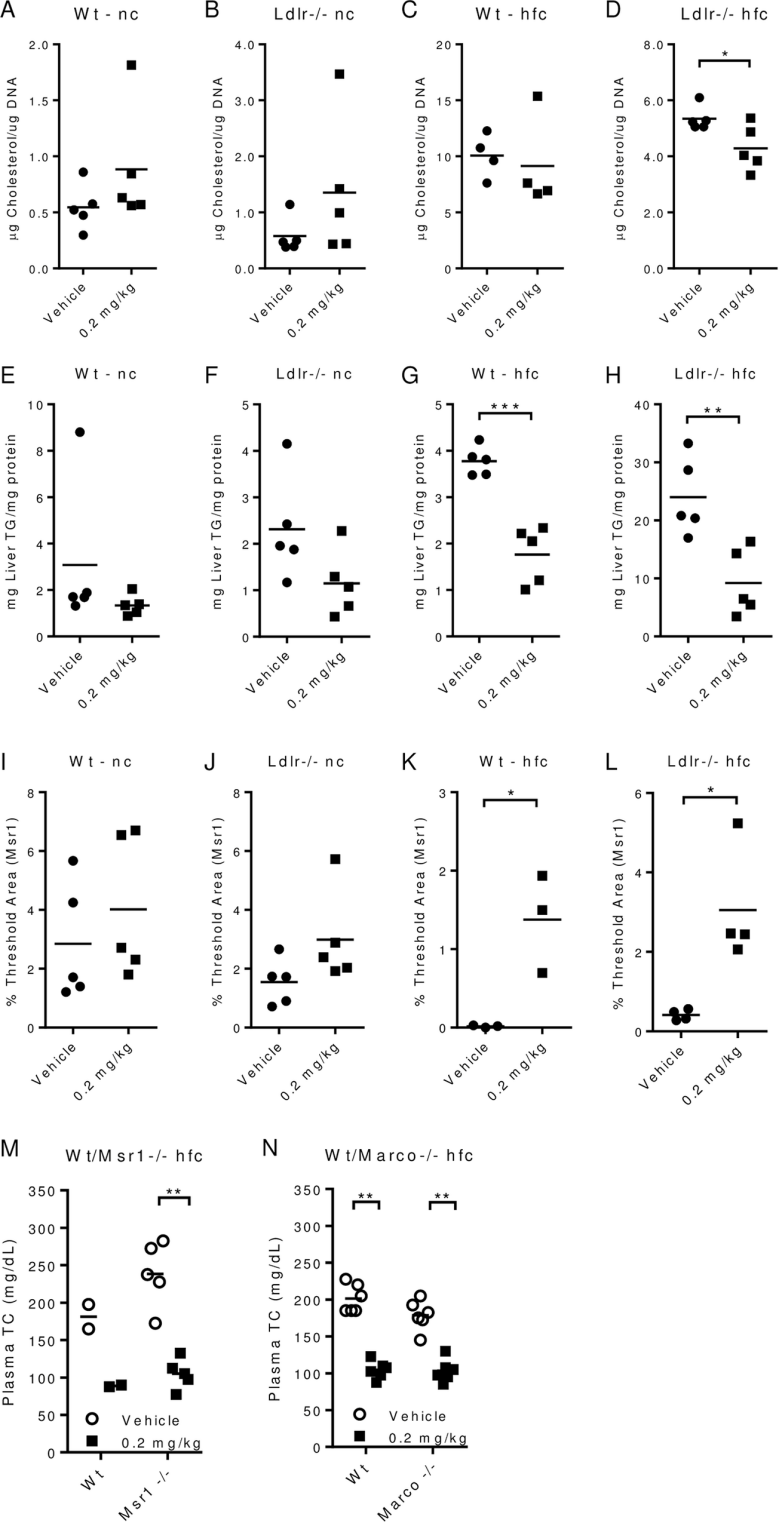
PEG-rMuIL-10 regulates cholesterol independent of scavenger receptors Msr1 and Marco. All mice were treated for 1–2 weeks by subcutaneous, daily (qd) injection with vehicle or 0.2 mg/kg PEG-rMuIL-10. Mice on nc or high fat chow hfc were pre-fed for 2 weeks prior to treatment. (A-D), liver tissue cholesterol quantitation from stated strain and diet, 5–10 animals per group with 4–5 livers randomly selected for quantitation. (E-H), liver tissue triglyceride quantitation was performed identically to cholesterol quantitation (A-D). (I-L), liver IHC Msr1 image quantitation from stated strain and diet. For quantitation of Msr1, 5–10 mice/cohort were initially analyzed and 3–5 mouse livers/cohort were randomly selected for signal quantification. (M), total plasma cholesterol from wt and Msr1-/- mice. (N), total plasma cholesterol from wt and Marco-/- mice. (A-L) statistics assessed by Students t-test where *p<0.05, **p<0.01, ***p<0.001. (M-N) statistics assessed by ANOVA multiple comparisons where *p<0.05, **p<0.01, ***p<0.001.

To further investigate PEG-rMuIL-10’s effect on the liver, we first assessed its regulation of liver fat, cholesterol and triglycerides. In confirmation of previous reports, PEG-rMuIL-10 treatment lowers total lipid concentrations in the liver (S7 Fig). PEG-rMuIL-10 treatment significantly lowers hepatic cholesterol of high fat fed Ldlr-/- mice (Fig 3A–3D). Hepatic triglyceride concentrations trend lower in wt and Ldlr-/- mice fed normal chow but are significantly lower in wt and Ldlr-/- mice fed high fat chow (Fig 3E–3H). Further examination revealed that PEG-rMuIL-10 treatment increased Ki67 (S8 Fig) and PCNA (S9–S12 Figs) expression. Additional immunohistochemistry analysis suggested these proliferating cells are Sca1 positive oval stem cells (data not shown). These data are consistent with IL-10’s ability to promote hepatic homeostasis [35, 36] but these effects do not consistently correlate with total cholesterol regulation. Proliferation and oval cell increases are the same in all strains and diets but wt type mice do not exhibit treatment associated cholesterol regulation. Previous reports suggest IL-10 regulates cholesterol through induction of scavenger receptor expression. Expression analysis revealed PEG-rMuIL-10 treatment induced changes in Msr1 and Marco expression independent of cholesterol reductions (S13–S16 Figs). However, IHC based quantitation revealed that upregulation of Msr1 appeared to trend with peripheral cholesterol regulation (Fig 3I–3L), where PEG-rMuIL-10 treatment of wt and Ldlr-/- mice fed high fat chow induced significant differences in detectible Msr1 (Fig 3K and 3L). Unexpectedly and contrary to expectations that IL-10 regulates cholesterol through induction of scavenger receptors [14], we discovered that PEG-rMuIL-10 mediated cholesterol reduction is independent of either Msr1 or Marco (Fig 3M and 3N).

### PEG-rMuIL-10 and AM0010 treatment increase peripheral and hepatic myeloid cells

**Fig 4:**
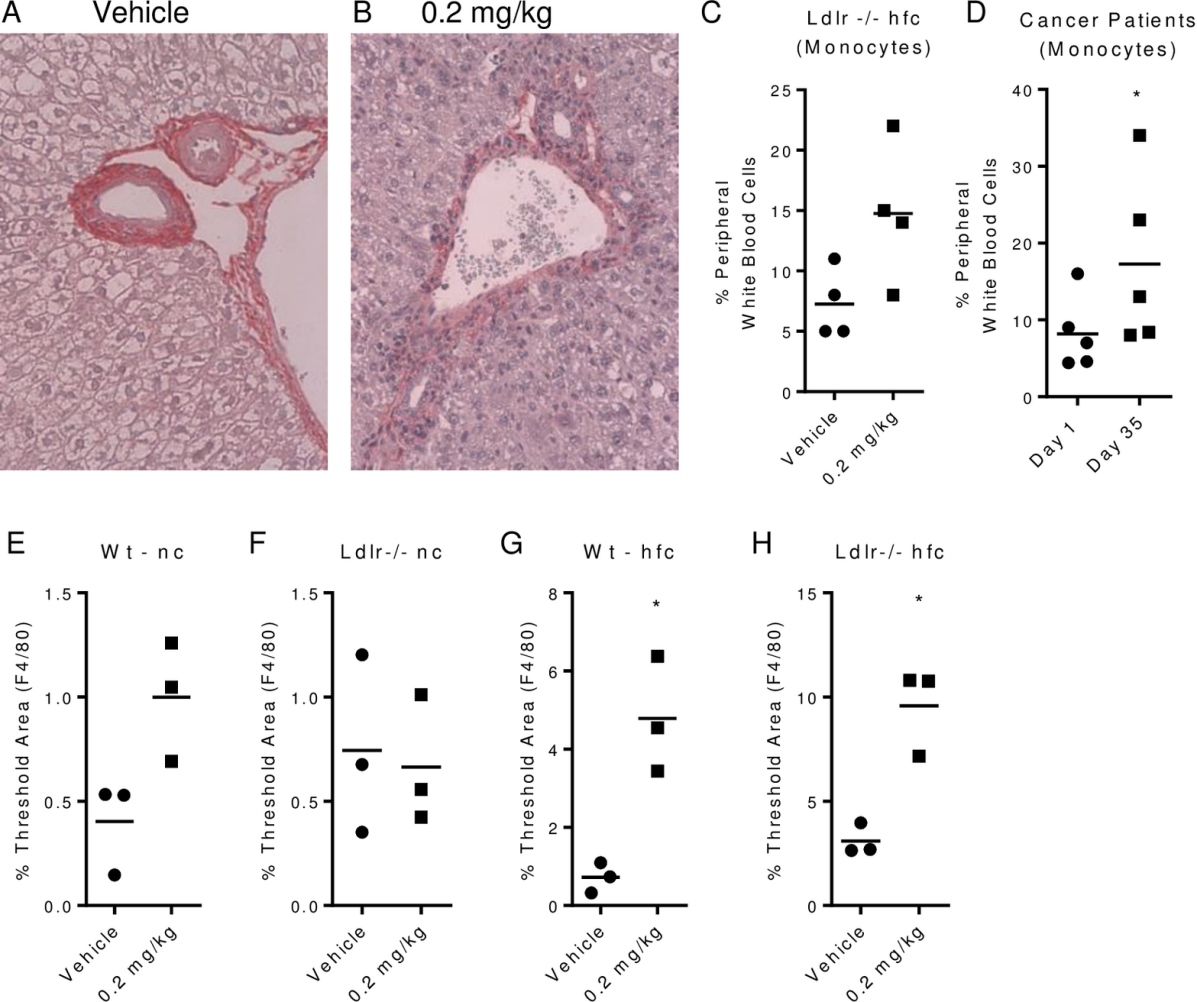
PEG-rMuIL-10 and AM0010 treatment increase peripheral and hepatic myeloid cells. All mice were treated for 1–2 weeks by s.c, qd injection with vehicle or 0.2 mg/kg PEG-rMuIL-10. Mice on nc or hfc were pre-fed for 2 weeks prior to treatment. (A-B), H&E periportal IHC same from (4A) vehicle or (4B) 0.2 mg/kg PEG-rMuIL-10 treated mouse. (C), quantitation of peripheral murine monocytes in Ldlr-/- hfc fed mice treated s.c. qd for two weeks with vehicle and 0.2 mg/kg PEG-rMuIL-10. (D), quantitation of peripheral human monocytes in cancer patients treated for 22 days s.c. qd with 20 μg/kg AM0010. (E-H), liver IHC F4/80 image quantitation from stated strain and diet. For quantitation of Msr1, 5–10 mice/cohort were initially analyzed and 3–5 mouse livers/cohort were randomly selected for signal quantification. Statistics were assessed by Student t test where *p<0.05.

We consistently observed a reduction in apparent steatotic cells (Fig 4A and 4B). Kupffer cells purportedly specifically regulate liver steatosis in response to high dietary fat intake [37] and therefore sit at the crossroads of dietary fat induced Fatty Liver Disease (FLD) and Nonalcoholic Steatohepatitis (NASH) [38]. Kupffer cells are macrophage/monocyte in origin [39]. In addition however, Kupffer cells also play a significant role in normal cholesterol uptake and catabolism. In early cholesterol regulation studies conducted with rabbits, Kupffer cells were reported to take up ~70% of total plasma LDL while constituting only ~4% of the cells in the liver [40]. In separate studies, Kupffer cells were shown to exhibit 18 fold greater cholesterol catabolism than hepatocytes [41]. We report, in agreement with Schering Plough data, (not shown), that mice and humans treated with PEG-rMuIL-10/AM0010 exhibit increased peripheral monocytes (Fig 4C and 4D). Similarly, CD14 and F4/80 hepatic expression moderately increases with treatment (S23–S26 Figs). F4/80 positive hepatic cells significantly increase with treatment only in high fat fed wt and Ldlr-/- mice (Fig 4E–4H) suggesting a role for these cells in response to both a high dietary fat diet and PEG-rMuIL-10 treatment. The reduction in steatotic cells prompted our investigation of whether PEG-rMuIL-10 treatment alters hepatic fibrosis. Long term high fat dietary treatment will lead to classic hexagonal collagen deposition within the liver [42]. We did not permit the study to proceed to this extent, however we did assess collagen deposition within the liver after 4 months of high fat diet treatment and 28 day treatment with PEG-rMuIL-10. Under these conditions the Ldlr-/- hfc mice exhibited substantially reduced periportal collagen deposition compared to controls (S20 Fig and S24 Fig). We further determined that PEG-rMuIL-10 treatment could reverse established periportal fibrosis (S25–S27 Figs). The periportal collagen levels are consistent with those observed in wt mice fed normal chow (S17 Fig compared to S27 Fig).

### AM0010 treatment enhances Kupffer cell phagocytosis of LDL and VLDL particles

**Fig 5:**
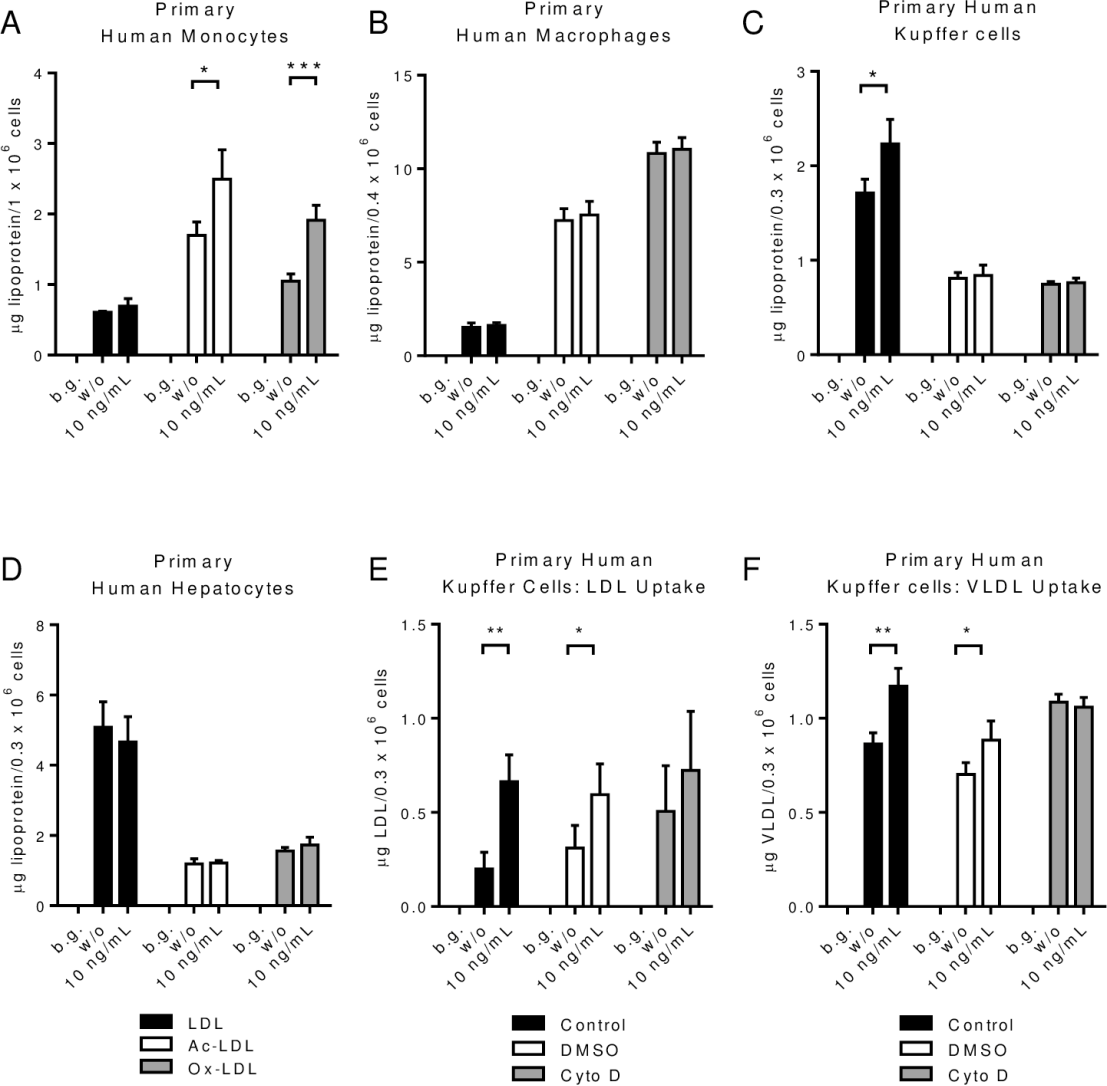
AM0010 treatment enhances Kupffer cell phagocytosis of LDL and VLDL particles. (A), primary human monocytes, (B), primary human macrophages, (C), primary human Kupffer cells and (D), human hepatocytes lipoprotein uptake with or without 24 hr. treatment of 100ng/mL PEG-rHuIL-10. (E-F) inhibition of LDL and VLDL uptake by Cytochalasin D. Each experiment represents 3–12 independent experiments. Background (b.g.) means cells without lipoprotein addition. Statistics for (A-F) were assessed by Students t-test where *p<0.05 and **p<0.01.

To specifically determine which cells in the liver respond to PEG-rIL-10, we treated primary human monocytes, macrophages, Kupffer cells and hepatocytes with AM0010 in vitro. Consistent with previous reports, AM0010 increased the uptake of Ac- and Ox-LDL, but not unmodified LDL by primary peripheral blood monocytes (Fig 5A). M-CSF differentiated macrophages did not respond to AM0010 (Fig 5B), whereas AM0010 increased primary Kupffer cell uptake of LDL but not Ac- or Ox-LDL (Fig 5C). Contrary to previous reports suggesting HepG2 liver tumor cells respond to IL-10 treatment by increased cholesterol uptake, primary hepatocyte uptake of unmodified, Ac- or Ox-LDL was unchanged in response to AM0010 (Fig 5D). Further investigation of this effect revealed that AM0010 treated human Kupffer cells take up more VLDL and that both LDL and VLDL uptake was blocked by Cytochalasin D, an inhibitor of phagocytosis (Fig 5E and 5F). While myeloid lineage cells are generally phagocytic, these data are the first to show that the nature of cholesterol uptake is different between monocytes, macrophages, and Kupffer cells in response to AM0010. To further substantiate the hypothesis that Kupffer cells are responsible for removal of plasma cholesterol we investigated whether there was an increase in efflux or canonical cholesterol catabolic pathways in vivo. PEG-rMuIL-10 treated mice do not exhibit discernable changes in fecal cholesterol (S28–S31 Figs), or fecal total bile acids (S32 Fig and S35 Fig). In addition, total serum bile acids did not change consistently with treatment in any groups (S36 Fig and S37 Fig). There was also no significant change to serum ApoA1 levels in Ldlr-/- mice fed normal chow (S40 Fig) or high fat fed wt and Ldlr-/- mice (S41 Fig and S42 Fig). Specific to liver cholesterol regulation, ApoB-100 levels were not changed in high fat fed Ldlr-/- mice (S43 Fig), the mice which exhibit the greatest decrease in plasma cholesterol (Fig 1G). These data suggest that PEG-rIL-10 regulates cholesterol via a non-canonical mechanism whereby significant amounts of serum cholesterol are removed firstly without the use of the Ldl receptor, and secondly via a means other than the induction of cholesterol uptake and catabolism by hepatocytes. Collectively these data suggest treatment with PEG-rMuIL-10 in vitro and AM0010 in vivo drives Kupffer cells to take up and remove plasma cholesterol.

### In vivo depletion of phagocytic cells abolishes PEG-rMuIL-10 reduction of cholesterol

**Fig 6:**
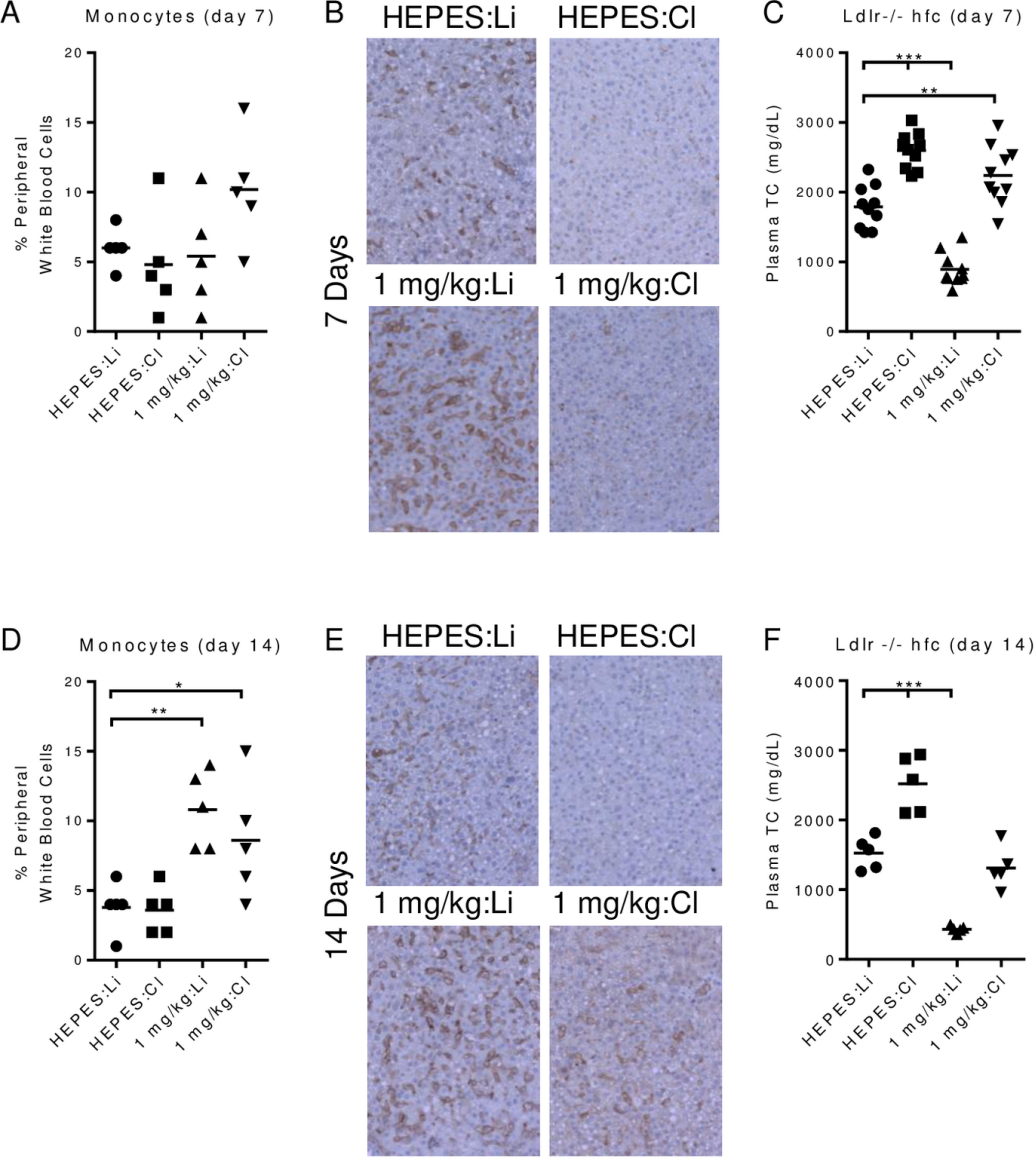
In vivo depletion of phagocytic cells abolishes PEG-rMuIL-10 reduction of cholesterol. Ldlr-/- or wt mice were fed nc or hfc for 1–2 weeks prior to initiation of stated treatment. Treatments were; 1 mg/kg PEG-rMuIL-10 (1 mg/kg), HEPES buffer (HEPES), 5 mg/ml clodronate liposomes (Cl) or liposomes (Li). (A), peripheral monocyte percentage of white blood cells of vehicle or PEG-rMuIL-10 treated mice in combination with clodronate liposomes or liposomes. (B), representative hepatic F4/80 images from mice in (A). (C), total plasma cholesterol after 7 days treatment with; HEPES/liposomes, HEPES/clodronate, PEG-rMuIL-10/liposomes, or PEG-rMuIL-10/clodronate. (D-F), the same series of experiments as in (A-C) after 14 days of treatment. (A-F) statistics were assessed by ANOVA multiple comparisons where *p<0.05, **p<0.01, ***p<0.001.

Given the in vitro effects of AM0010, we hypothesized that phagocytic cells within the myeloid lineage were responsible for PEG-rMuIL-10s control of plasma cholesterol. We used a standard technique for removing phagocytic Kupffer cells [43] by dosing animals with clodronate liposomes in the presence or absence of PEG-rMuIL-10. We tracked the changes in peripheral monocytes (Fig 6A) versus changes in F4/80 cells within the liver (Fig 6B). We also confirmed hepatocyte health by TUNEL IHC (no increase in apoptotic cells), and H&E (no change in the histological morphology or cellular organization), both data not shown. We chose to dose with 1 mg/kg instead of the typical 0.2 mg/kg PEG-rMuIL-10 dose to ensure sufficient therapeutic compound was available to induce hypothesized functional changes to all of the myeloid lineage cells within the animal, in context of treatment with liposome encapsulated clodronate. Peripheral monocyte numbers were not reduced by liposome encapsulated clodronate treatment (Fig 6A); HEPES + Clodronate (HEPES:Cl). 7 days of dosing with PEG-rMuIL-10 does not yet change peripheral monocyte levels in the control; 1 mg/kg PEG-rMuIL-10 + Liposome (1 mg/kg:Li) but does cause a detectible increase in the 1 mg/kg PEG-rMuIL-10 + Clodronate (1 mg/kg:Cl) group. Interestingly, after 7 days of treatment, the liver was depleted of detectible F4/80 positive cells, even in the 1 mg/kg:Cl group (Fig 6B). This indicates that while the peripheral monocyte population varied from slightly increased to unchanged by PEG-rMuIL-10 dosing, the liver Kupffer cells were depleted after 7 days of treatment in the same animals. Depletion of liver Kupffer cells in the HEPES + Clodronate group, (HEPES/Cl) increased total serum cholesterol as did concomitant treatment with PEG-rMuIL10 and Clodronate Liposomes (Fig 6C). Mice exhibiting depleted hepatic Kupffer cells but increased serum monocytes were non-responsive to PEG-rMuIL-10 treatment; 1 mg/kg PEG-rMuIL-10 + Liposome (1 mg/kg:Li) vs. 1 mg/kg PEG-rMuIL-10 + Clodronate (1 mg/kg:Cl), (Fig 6C). These data confirm the in vitro analysis that PEG-rIL-10 does not directly induce the uptake of any form of cholesterol by hepatocytes. Moreover, the loss of Kupffer cells led to an increase in total peripheral cholesterol, even in the presence of normal peripheral monocyte numbers; (Fig 6A–6C) (HEPES:Cl). After 14 days of treatment, PEG-rMuIL-10 treated mice exhibited statistically increased peripheral monocytes; 1 mg/kg PEG-rMuIL-10 + Liposome (1 mg/kg:Li) and 1 mg/kg PEG-rMuIL-10 + Clodronate (1 mg/kg:Cl) (Fig 6D). The livers of mice treated with 1 mg/kg PEG-rMuIL-10 + Clodronate (1 mg/kg:Cl) were just beginning to show detectible F4/80 positive cells (Fig 6E). As a result, the mice with increased hepatic F4/80 positive cells (1mg/kg:Cl) exhibited partially restored sensitivity to PEG-rMuIL-10 cholesterol lowering (Fig 6F). These data suggest PEG-rMuIL-10 may facilitate Kupffer cell liver homeostasis, by overcoming the effects of clodronate and enhancing the repopulation of Kupffer cells in the liver thereby reestablishing their ability to lower cholesterol.

## Results and Discussion

Therapeutic control of systemic cholesterol has predominantly focused on inhibiting dietary uptake and/or inhibition of endogenous synthesis. Ezetimibe inhibits dietary uptake [44, 45]. Statins block hepatocyte cholesterol synthesis by inhibiting the HMG-CoA pathway [46]. Recently, significant progress has been made in blocking PCSK9, a co-factor involved in regulating the recycling of the LDL receptor [47]. Blockade of PCSK9 increases the rate at which LDL particles are removed from the blood [48]. Kynamro is an antisense oligonucleotide that hybridizes with hepatocyte specific apoB-100 [49], but treatment leads to fatty liver disease [50]. Juxtapid predominantly inhibits microsomal triglyceride transfer protein in the liver [51, 52], but leads to fat accumulation in the liver [53]. Our findings add to recent data [54, 55] that macrophages actively participate in cholesterol regulation via remodeling atherosclerotic plaques and reverse cholesterol transport [56]. However, our data suggest that the liver resident Kupffer cells are significantly involved in cholesterol regulation and can be therapeutically engaged by treatment with AM0010. Kupffer cells represent 80–90% of all tissue resident macrophages in the body [57] and make up approximately 10–15% of the total 10–30 billion cells in an adult liver [58]. Kupffer cells are 18 times more efficient in cholesterol catabolism than hepatocytes and have been shown to take up ~70% [59] of plasma LDL and 30% [60] of total plasma cholesterol in rats and rabbits respectively. While Kupffer cells have been implicated in both fatty liver disease (FLD) and non-alcoholic steatohepatitis (NASH), our data suggests that the function of the Kupffer cells, rather than the absolute number may be dysfunctional in these diseases. This hypothesis is augmented by reports that Kupffer cells take up a substantial amount of serum cholesterol while constituting a small percentage of total cells in the liver, and that Kupffer cells catabolize this cholesterol at a higher rate than hepatocytes. It therefore seems possible that in diseased states, Kupffer cells may exhibit catabolic dysfunctions. The catabolism of lipid stores by lysosomal degradative pathways is termed lipophagy [61]. This is a growing field of investigation and there are no reports on Kupffer cells utilizing this pathway to catabolize cholesterol. Given the data presented herein, we hypothesize that Kupffer cells may naturally uptake and catabolize plasma cholesterol by lipophagy. Further to this hypothesis, we also show here that treatment of hypercholesterolemic mice with PEG-rIL-10 significantly lowers overall hepatic lipid and cholesterol levels leading to reduced steatosis. The reduction in steatotic cells is concomitant with the reversal of periportal collagen deposition and associated fibrosis. These data imply that chronic treatment with PEG-rIL-10 may significantly reduce NASH by directly engaging Kupffer cells to uptake and catabolize plasma cholesterol, rather than elicit the inappropriate accumulation of lipids by hepatocytes. Kupffer cell expression of TNFα is thought to be causative in this process [62]. IL-10 is a potent suppressor of inflammation associated elevated TNFα levels [63]. These data are in keeping with both preclinical [64] and clinical [65] data that rIL-10 treatment reduces liver fibrosis. Both Schering Plough and our clinical data suggest chronic exposure to rHuIL-10/AM0010 is safe and well tolerated. It is therefore possible that in addition to lowering cholesterol in general, AM0010 may represent an alternative treatment for people suffering from NASH.

## Conclusions

This is the first report to our knowledge to link IL-10’s regulation of Kupffer cell phagocytosis to the decrease in peripheral cholesterol. The phagocytic myeloid immune system, including Kupffer cells, may represent a therapeutically untapped arm of the immune system. When properly engaged, this endogenous system appears to lower total plasma cholesterol levels without increasing inappropriate liver accumulation of cholesterol or triglycerides.

## Materials and Methods

### Serum and tissue cholesterol and chemistry quantitation

25μL aliquots of ground mouse livers were used for the extraction of cholesterol/cholesteryl esters using a cholesterol/cholesteryl assay kit (BioVision) according to the manufacturer’s instructions and measured with a Spectra Max 340 PC (Molecular Devices). Genomic DNA was extracted from equal amounts of sample using a DNeasy kit (QIAGEN) and measured with a NanoVue Plus (GE Healthcare) to normalize the cholesterol/cholesteryl measurements. Serum LDL-C or HDL-C quantitation was performed on a Beckman Coulter AU System LDL-Cholesterol test using a two reagent homogenous system at a 1:300 dilution (IDEXX). Plasma chemistry was performed on a Catalyst Chemistry Analyzer (IDEXX). Lipoproteins were separated by gel filtration chromatography via Superose 6 resin, at room temperature. VLDL, LDL and HDL controls were used to determine column residence time. Feces were collected from denoted strain and diet fed mice, dried o/n at 100°C and cholesterol was re-suspended in a ratio of 7:11:0.1, chrolophorm:isopropanol:NP-40 and incubated while vortexing at RT for 30 minutes and after centrifugation, supernatants were collected 3X. The supernatant was evaporated o/n and residue was re-suspended in cholesterol assay kit buffer. Feces were collected and dried at 100°C o/n. Total bile acids were collected via resuspension of feces in 75% ethanol. This was incubated with vortexing at RT for 1 hr. and then after centrifugation, the supernatant was taken and assayed in accordance with Diazyme total bile acids kit instructions.

#### Tissue triglyceride quantitation

Mouse livers were ground up in 200 **μ**L water with pestles (VWR). 25**μ**L aliquots were used for the extraction of triglycerides using a Triglyceride Assay kit (Biovision) according to the manufacturer’s instructions and measured with a Spectra Max 340 PC (Molecular Devices). Protein concentration was determined using the Pierce BCA protein assay (Thermo Scientific) according to the manufacturer’s instructions and measured with a Spectra Max 340 PC.

### qPCR analysis

Mouse livers were ground up in Buffer RLT (QIAGEN) with 10 μL β-mercaptoethanol (Sigma-Aldrich) using pestles (VWR), after which RNA was extracted using an RNeasy kit (QIAGEN) according to the manufacturer’s instructions. The purified RNA was used as template for RT-PCR using an RT2 First Strand kit (QIAGEN). One microliter aliquots of the resulting cDNA samples were used for qPCR of the indicated transcripts on an ABI PRISM 7700 Sequence Detection System or an ABI ViiA 7 Real Time PCR Machine (Life Technologies). CT values were normalized to the average CT value of ***Gapdh*** and ***Gusb***. All primers used were purchased from QIAGEN.

### Immunohistochemistry

Liver tissues were fixed with 10% neutral-buffered formaldehyde and were embedded in paraffin. Tissue specimens were cut into 5-μm-thick sections, de-paraffinized in xylene sections, and hydrated in a graded series of alcohol solutions (100%, 95%, 80%, 70%, 50%– 3 changes –5 minutes each). The tissues on slides underwent heat-induced epitope retrieval (10 mmol/L sodium citrate buffer at 98°C for 20 mins), then treated with 3% H_2_O_2_ to quench endogenous peroxidase. Sections were incubated in blocking solution (5% neutral goat serum) for 1 hr., at rt. Primary antibodies of choice were applied on the slides and incubated in humid chamber overnight at 4°C. Secondary biotinylated antibody was then applied at 1:250 dilution (Vector Lab, Burlingame, CA, USA), followed by incubation with streptavidin peroxidase. Sections were washed with phosphate buffer saline (PBS) 3X after each step. Sections were stained with DAB substrate and counterstained with Mayer's hematoxylin for 2 mins. Slides were dehydrated in 3 changes of 100% ethanol, cleared, and mounted.

#### Anti-F4/80, Anti-Msr1 and PCNA

Liver tissues were fixed with 10% neutral-buffered formaldehyde and were embedded in paraffin. Tissue specimens were cut into 5-**μ**m-thick sections, deparaffinized in xylene sections, and were hydrated in a graded series of alcohol solutions (100%, 95%, 80%, 70%, 50%–three changes –5 minutes each). The tissues on slides underwent heat-induced epitope retrieval (10 mmol/L sodium citrate buffer at 98°C for 20 minutes), then treated with 3% H_2_O_2_ to quench endogenous peroxidase. Sections were incubated in blocking solution (5% neutral goat serum) for 1 hour at room temperature. Primary antibodies of choice were applied on the slides and incubated in humid chamber overnight at 4°C. Secondary biotinylated antibody was then applied at 1:250 dilution (Vector Lab, Burlingame, CA, USA), followed by incubation with streptavidin peroxidase. Sections were washed with phosphate buffer saline (PBS) three times after each step. Sections were stained with DAB substrate and counterstained with Mayer's hematoxylin for 2 minutes. Slides were dehydrated in three changes of 100% ethanol, cleared, and mounted.

#### Picosirius red staining

Slides were heated in an oven at 60°C for 45 minutes, deparaffinized using xylene and series of alcohols and rehydrated in water; then kept for 60 minutes in freshly prepared Picosirius red solution according to manufacturer’s instructions, followed by two washes in acidified water. Nuclei were stained with Weigert’s hematoxylin for 8–10 minutes, dehydrated in three changes of 100% ethanol, cleared, and mounted.

#### Image quantitation

PEG-rMuIL-10 treated livers were compared to vehicle-treated livers, 2–5 mice per group were randomly selected and stained with Sirius Red (Polyscience Inc.), anti-PCNA (Abcam), Hematoxylin (American MasterTech), anti-Msr1 (Abcam), anti-F4/80 (Abcam). For each liver, 8–10 independent images were collected using the 20 x objective. An average area of signal was then analyzed using MetaMorph Imaging Software (Molecular Devices) by applying a color threshold on a representative field and adjusting the pixel distribution to correspond with a positive signal. All images were taken with a 20X objective.

### In vitro uptake assays

Monocytes were isolated from Ficoll centrifugation isolated PBMC by Miltenyi magnetic bead positive selection. Macrophages were differentiated from positively selected peripheral blood monocytes with 50ng/mL GM-CSF (BioLegend) in cRPMI for 7 days. Human primary hepatocytes (Triangle Research Labs) and Kupffer cells (Invitrogen), were thawed and plated in 24-well or 96-well plates and incubated o/n in hepatocyte incubation medium (phenol-red free RPMI, pen/strep, Cell Maintenance Supplement B (Invitrogen)). Cells were washed and exposed for 24 hrs to AM0010. Cells were washed once and exposed to 15–20 μl DiI-LDL, DiI-VLDL, DiI-OxLDL or DiI-AcLDL, (Alfa Aesar), 2μl DMSO, 15μM Cytochalasin D (Sigma), where uptake was measured after 4 hours. All cells were washed once in 1XPBS and lysed with 110 μl cell lysis buffer (Sigma-Aldrich). 45 μl of cell lysate was transferred to clear bottom black walled plates (Greiner Bio-One) where fluorescence was read at 575 nm.

### In vivo studies

Aragen Biosciences conducted mouse studies in accordance with standard operating procedures and established guidelines approved by their Institutional Animal Care and Use Committee (IACUC), which is called the Aragen IACUC committee. Aragen Bioscience http://www.aragenbio.com/. 380 Woodview Ave. Morgan Hill, CA 95037.

#### Hypercholesterolemia model

In-life portions of the studies were performed at Aragen Biosciences. Wt and Ldlr-/- C57BL/6 mice (7–8 weeks old), from The Jackson Laboratory, were maintained on normal chow or fed High Fat Chow (HFC, 0.2% Cholesterol, 21% Fat) diet for 2 or 7 weeks prior to dosing. PEGylated recombinant mouse IL-10 (PEG-rMuIL-10) or vehicle (10 mM HEPES, 100 mM NaCl, pH 6.5, 0.05% mouse serum albumin) was dosed subcutaneously daily for 2 to 3 weeks; mice were maintained on their respective diets throughout dosing. Msr1-/- (The Jackson Laboratory) and Marco-/- (a kind gift from Dr. Lester Kobzik) mice were treated similarly as above. For clodronate depletion studies, mice were dosed, intravenously, with clodronate liposomes (5mg/ml clodronate) or vehicle liposomes suspended in 1X PBS, every three days (first dose: 0.2 ml, subsequent doses: 0.1 ml), starting one day before PEG-rMuIL-10 dosing. Monocytes were quantified with a ProCyte Dx Hematology Analyzer. Both ezetimibe (75mg/kg) and simvastatin (10mg/kg) were administered by oral gavage daily.

### Clinical studies

Fully consented male and female patients, 18–80 years old suffering from terminal, surgically non-operable, late stage neoplastic disease of non-lymphatic origin, were subjected to weekly peripheral blood collection by trained phlebotomists under IRB approved protocol; FDA study ID# NCT02009449. Serum cholesterol quantitation was performed by local clinical laboratories following standard procedures. AM0010 was administered at 1, 2.5, 5, 10, or 20 μg/kg s.c. qd. For the Schering Plough studies, IL-10 was administered as stated in tables. Samples for serum chemistry analysis from the rHuIL-10 safety study, (Schering Plough Study Protocol #C97-070) and Crohn’s study (Schering Plough Study Protocol #C195-152) were obtained from fully consented male and female patients, 18–60 years old, and analyzed in a similar manner to the ARMO studies. All patients provided written consent and the record of their consent is contained within the Trial Master File. Three IRBs approved the site specific protocols for the administration of AM0010 to cancer patients. These IRBs are as follows:

Dana-Farber/Harvard Cancer Center: DFCI IRB

Memorial Sloan Kettering Cancer Center: Memorial Sloan Kettering Cancer Center IRB

MD Anderson Cancer Center: MD Anderson Cancer Center IRB

## Supporting Information

S1 Fig Related to Fig 2, PEG-rMuIL-10 treatment effect on scavenger receptor regulation in the Ileum. S1 Fig ileum mRNA expression profiling of Ldlr-/- fed high fat diet for 2 weeks and treated s.c. daily with 0.2 mg/kg PEG-rMuIL-10 for 2 weeks.
(JPG)

S2 Fig Related to Fig 2, PEG-rMuIL-10 treatment effect on scavenger receptor regulation in the Ileum. S2 Fig jejunum mRNA expression profiling of Ldlr-/- fed high fat diet for 2 weeks and treated s.c. daily with 0.2 mg/kg PEG-rMuIL-10 for 2 weeks.
(JPG)

S3 Fig Related to Fig 2, PEG-rMuIL-10 effect on the Mevalonate pathway. S3–S6 Figs, Expression of Mevalonate Pathway genes: 3-hydroxy-3-methyl-glutaryl-CoA reductase (Hmgcr), 3-hydroxyl-3-methyl-glutaryl-CoA Synthase 1 (Hmgcs1) and 3-hydroxyl-3-methyl-glutaryl-CoA Synthase 2 (Hmgcs2) were assessed by qPCR at the end of treatment. S3 Fig, wt mice on nc, were treated for 1–2 weeks with vehicle or 0.2 mg/kg s.c. qd PEG-rMuIL-10.
(JPG)

S4 Fig Related to Fig 2, PEG-rMuIL-10 effect on the Mevalonate pathway. S4 Fig, Ldlr-/- mice on nc, were treated for 1–2 weeks with vehicle or 0.2 mg/kg s.c. qd PEG-rMuIL-10.
(JPG)

S5 Fig Related to Fig 2, PEG-rMuIL-10 effect on the Mevalonate pathway. S5 Fig, wt mice mice on high fat chow (hfc) were fed for 2 weeks and treated for two weeks with vehicle or 0.2 mg/kg s.c. qd PEG-rMuIL-10.
(JPG)

S6 Fig Related to Fig 2, PEG-rMuIL-10 effect on the Mevalonate pathway. S6 Fig, Ldlr-/- mice on high fat chow (hfc) were fed for 2 weeks and treated for two weeks with vehicle or 0.2 mg/kg s.c. qd PEG-rMuIL-10.
(JPG)

S7 Fig Related to Fig 3, PEG-rMuIL-10 treatment reduces hepatic lipids. S7 Fig, Ldlr-/- mice were fed hfc for 2 weeks the dosed s.c. daily with 0.2 mg/kg PEG-rMuIL-10 for 2 weeks. 10–20 images per mouse were quantified with 4 mice per cohort randomly selected. The median percent threshold of signal was determined and plotted for each mouse. Wt or Ldlr-/- mice on nc were treated for 1–2 weeks with vehicle or 0.2 mg/kg s.c. qd PEG-rMuIL-10. Wt or Ldlr-/- mice on high fat chow (hfc) were fed for 4 weeks and treated with vehicle or 0.2 mg/kg s.c. qd PEG-rMuIL-10 during the last 2 weeks. Statistics assessed by Students T test where *p<0.05, **p<0.01, ***p<0.001.
(JPG)

S8 Fig Related to Fig 3, PEG-rMuIL-10 treatment induces hepatic proliferation. S8 Fig, hepatic expression analysis of Ki67 from 5–10 wt or LDLR-/- mice per group fed nc or hfc.
(JPG)

S9 Fig Related to Fig 3, PEG-rMuIL-10 treatment induces hepatic proliferation. S9–S12 Figs, Liver PCNA IHC. 10–20 liver images per mouse were quantified with 2–3 mice per cohort randomly selected. The median percent threshold of signal was determined and plotted for each mouse. PCNA IHC image quantitation of wt mice on nc, S9 Fig, wt mice on nc. Statistics assessed by Students T test where *p<0.05, **p<0.01, ***p<0.001.
(JPG)

S10 Fig Related to Fig 3, PEG-rMuIL-10 treatment induces hepatic proliferation. S10 Fig, Ldlr-/- mice fed nc. Statistics assessed by Students T test where *p<0.05, **p<0.01, ***p<0.001.
(JPG)

S11 Fig Related to Fig 3, PEG-rMuIL-10 treatment induces hepatic proliferation. S11 Fig, wt mice on nc. Statistics assessed by Students T test where *p<0.05, **p<0.01, ***p<0.001.
(JPG)

S12 Fig Related to Fig 3, PEG-rMuIL-10 treatment induces hepatic proliferation. S12 Fig, Ldrl-/- on hfc. Statistics assessed by Students T test where *p<0.05, **p<0.01, ***p<0.001.
(JPG)

S13 Fig Related to Fig 3, PEG-rMuIL-10 treatment induces hepatic proliferation. S13–S16 Figs, liver expression analysis of Pcsk9, CD14, F4/80, Msr1 and Marco genes from wt mice fed nc, S13 Fig, wt mice fed nc treated with vehicle or 0.2 mg/kg s.c. qd PEG-rMuIL-10.
(JPG)

S14 Fig Related to Fig 3, PEG-rMuIL-10 treatment induces hepatic proliferation. S14 Fig, Ldlr-/- mice fed nc treated with vehicle or 0.2 mg/kg s.c. qd PEG-rMuIL-10.
(JPG)

S15 Fig Related to Fig 3, PEG-rMuIL-10 treatment induces hepatic proliferation. S15 Fig, wt mice fed hfc treated with vehicle or 0.2 mg/kg s.c. qd PEG-rMuIL-10.
(JPG)

S16 Fig Related to Fig 3, PEG-rMuIL-10 treatment induces hepatic proliferation. S16 Fig, Ldlr-/- mice fed hfc treated with vehicle or 0.2 mg/kg s.c. qd PEG-rMuIL-10.
(JPG)

S17 Fig Related to Fig 4, PEG-rMuIL-10 treatment reduces liver fibrosis. Liver Sirius Red IHC. S17–S20 Figs, representative periportal liver images of vehicle dosed wt and Ldlr-/- mice on nc and hfc. S21–S24 Figs, representative periportal images of 0.2 mg/kg s.c. qd PEG-rMuIL-10 dosed wt and LDLR-/- mice fed nc and hfc. S17 Fig, wt fed nc, vehicle treated for 1 week.
(JPG)

S18 Fig Related to Fig 4, PEG-rMuIL-10 treatment reduces liver fibrosis. S18 Fig, Ldlr-/- fed nc, vehicle treated for 1 week.
(JPG)

S19 Fig Related to Fig 4, PEG-rMuIL-10 treatment reduces liver fibrosis. S19 Fig, wt on hfc, vehicle treated for 2 weeks.
(JPG)

S20 Fig Related to Fig 4, PEG-rMuIL-10 treatment reduces liver fibrosis. S20 Fig, Ldlr-/- fed hfc, vehicle treated for 2 weeks.
(JPG)

S21 Fig Related to Fig 4, PEG-rMuIL-10 treatment reduces liver fibrosis. S21 Fig, wt fed nc, PEG-rMuIL-10 treated for 1 week.
(JPG)

S22 Fig Related to Fig 4, PEG-rMuIL-10 treatment reduces liver fibrosis. S22 Fig, Ldlr-/- fed nc, PEG-rMuIL-10 treated for 1 week.
(JPG)

S23 Fig Related to Fig 4, PEG-rMuIL-10 treatment reduces liver fibrosis. S23 Fig, wt fed hfc for 4 weeks, treated with PEG-rMuIL-10 for 2 weeks.
(JPG)

S24 Fig Related to Fig 4, PEG-rMuIL-10 treatment reduces liver fibrosis. S24 Fig, Ldlr-/- fed hfc for 4 weeks, treated with PEG-rMuIL-10 for 2 weeks.
(JPG)

S25 Fig Related to Fig 4, PEG-rMuIL-10 treatment reduces liver fibrosis. S25Fig, Ldlr-/- fed hfc for 7 weeks.
(JPG)

S26 Fig Related to Fig 4, PEG-rMuIL-10 treatment reduces liver fibrosis. S26 Fig, Ldlr-/- from S25 Fig, dosed with vehicle for 3 weeks while remaining on hfc.
(JPG)

S27 Fig Related to Fig 4, PEG-rMuIL-10 treatment reduces liver fibrosis. S27 Fig, Ldlr-/- from S25 Fig, dosed with 0.2mg/kg for 3 weeks with 0.2mg/kg PEG-rMuIL-10 s.c. qd while remaining on hfc.
(JPG)

S28 Fig Related to Fig 5, PEG-rMuIL-10 treatment does not increase canonical cholesterol catabolism. Mice treated as in S28–S31 Figs. Fecal bile acids and cholesterol measurements represent the total fecal matter from 2 weeks of dosing from two separate cages housing 4–5 mice per cage. To ensure sufficient material to quantify after drying and extraction, the total fecal material from each cage had to be pooled. S28 Fig fecal cholesterol of wt mice fed nc.
(JPG)

S29 Fig Related to Fig 5, PEG-rMuIL-10 treatment does not increase canonical cholesterol catabolism. S29 Fig, fecal cholesterol of Ldlr-/- mice fed nc.
(JPG)

S30 Fig Related to Fig 5, PEG-rMuIL-10 treatment does not increase canonical cholesterol catabolism. S30 Fig, fecal cholesterol of wt and mice fed high fat chow (hfc).
(JPG)

S31 Fig Related to Fig 5, PEG-rMuIL-10 treatment does not increase canonical cholesterol catabolism. S31 Fig, fecal cholesterol of Ldlr-/- mice fed high fat chow (hfc).
(JPG)

S32 Fig Related to Fig 5, PEG-rMuIL-10 treatment does not increase canonical cholesterol catabolism. S32 Fig, fecal bile acids of wt mice fed nc.
(JPG)

S33 Fig Related to Fig 5, PEG-rMuIL-10 treatment does not increase canonical cholesterol catabolism. S33 Fig, fecal bile acids of wt and Ldlr-/- mice fed nc.
(JPG)

S34 Fig Related to Fig 5, PEG-rMuIL-10 treatment does not increase canonical cholesterol catabolism. S34 Fig, fecal bile acids of wt mice fed hfc.
(JPG)

S35 Fig Related to Fig 5, PEG-rMuIL-10 treatment does not increase canonical cholesterol catabolism. S35 Fig, fecal bile acids of Ldlr-/- mice fed hfc.
(JPG)

S36 Fig Related to Fig 5, PEG-rMuIL-10 treatment does not increase canonical cholesterol catabolism. S36 Fig, serum bile acids in wt mice fed nc.
(JPG)

S37 Fig Related to Fig 5, PEG-rMuIL-10 treatment does not increase canonical cholesterol catabolism. S37 Fig, serum bile acids in Ldlr-/- mice fed nc.
(JPG)

S38 Fig Related to Fig 5, PEG-rMuIL-10 treatment does not increase canonical cholesterol catabolism. S38 Fig, serum bile acids in wt mice fed hfc.
(JPG)

S39 Fig Related to Fig 5, PEG-rMuIL-10 treatment does not increase canonical cholesterol catabolism. S39 Fig, serum bile acids in Ldlr-/- mice fed hfc.
(JPG)

S40 Fig Related to Fig 5, PEG-rMuIL-10 treatment does not increase canonical cholesterol catabolism. S40 Fig serum ApoA1 in Ldlr-/- mice fed nc.
(JPG)

S41 Fig Related to Fig 5, PEG-rMuIL-10 treatment does not increase canonical cholesterol catabolism. S41 Fig, serum ApoA1 in wt mice fed hfc.
(JPG)

S42 Fig Related to Fig 5, PEG-rMuIL-10 treatment does not increase canonical cholesterol catabolism. S42 Fig, serum ApoA1 in Ldlr-/-mice fed hfc.
(JPG)

S43 Fig Related to Fig 5, PEG-rMuIL-10 treatment does not increase canonical cholesterol catabolism. S43 Fig, serum ApoB-100 in Ldlr-/- mice fed hfc.
(JPG)

S1 Table Related to Fig 1, PEG-rIL-10 treatment is safe and reduces plasma cholesterol levels. S1 Table, Serum chemistry data from Schering Plough safety study in health volunteers. Cholesterol, LDL, HDL, AopA and ApoB levels from 12 heathy donors treated subcutaneous (s.c.), every other day with 8 μg/kg rHuIL-10 for 11 days. Serum chemistry was analyzed pre-dose and on day 12.
(JPG)

S2 Table Related to Fig 1, PEG-rIL-10 treatment is safe and reduces plasma cholesterol levels. S2 Table, Serum chemistry data from Schering Plough Crohn’s study. 263 total patients with active steroid refractory Crohn’s disease were treated s.c., daily (qd), for 28 days with 1, 4, 8, 20 μg/kg rHuIL-10 or placebo. S2 Table lists serum chemistry markers and the number of patients who exhibited changes in these markers at any time during the study.
(JPG)

S3 Table Related to Fig 1, PEG-rIL-10 treatment is safe and reduces plasma cholesterol levels. S3 Table, Serum chemistry data was pooled from all 7 PEG-rMuIL-10 dosing studies. S3 Table illustrates the plasma/serum levels after dosing with 0.2 mg/kg PEG-rMuIL-10 for listed liver function markers.
(JPG)

## References

[1] Chang J, Kunkel SL, Chang CH. Negative regulation of MyD88-dependent signaling by IL-10 in dendritic cells. Proceedings of the National Academy of Sciences of the United States of America. 2009;106(43):18327–32. 10.1073/pnas.0905815106 19815506 PMC2775313

[2] Curtale G, Mirolo M, Renzi TA, Rossato M, Bazzoni F, Locati M. Negative regulation of Toll-like receptor 4 signaling by IL-10-dependent microRNA-146b. Proceedings of the National Academy of Sciences of the United States of America. 2013;110(28):11499–504. 10.1073/pnas.1219852110 23798430 PMC3710884

[3] Qasimi P, Ming-Lum A, Ghanipour A, Ong CJ, Cox ME, Ihle J, et al Divergent mechanisms utilized by SOCS3 to mediate interleukin-10 inhibition of tumor necrosis factor alpha and nitric oxide production by macrophages. The Journal of biological chemistry. 2006;281(10):6316–24. 10.1074/jbc.M508608200 . 16352613

[4] Chan IH, Wu V, Bilardello M, Mar E, Oft M, Van Vlasselaer P, et al The Potentiation of IFN-gamma and Induction of Cytotoxic Proteins by Pegylated IL-10 in Human CD8 T Cells. J Interferon Cytokine Res. 2015 10.1089/jir.2014.0221 . 26309093

[5] Mumm JB, Emmerich J, Zhang X, Chan I, Wu L, Mauze S, et al IL-10 elicits IFNgamma-dependent tumor immune surveillance. Cancer cell. 2011;20(6):781–96. 10.1016/j.ccr.2011.11.003 . 22172723

[6] Infante JR, Naing A, Papadopoulos KP, Autio KA, Ott PA, Wong DJL, et al A first-in-human dose escalation study of PEGylated recombinant human IL-10 (AM0010) in advanced solid tumors. ASCO Meeting Abstracts. 2015;33(15_suppl):3017.

[7] Nagalakshmi ML, Murphy E, McClanahan T, de Waal Malefyt R. Expression patterns of IL-10 ligand and receptor gene families provide leads for biological characterization. International immunopharmacology. 2004;4(5):577–92. 10.1016/j.intimp.2004.01.007 . 15120644

[8] Denning TL, Campbell NA, Song F, Garofalo RP, Klimpel GR, Reyes VE, et al Expression of IL-10 receptors on epithelial cells from the murine small and large intestine. International immunology. 2000;12(2):133–9. . 10653848 10.1093/intimm/12.2.133

[9] Ritchlin C, Haas-Smith SA. Expression of interleukin 10 mRNA and protein by synovial fibroblastoid cells. The Journal of rheumatology. 2001;28(4):698–705. . 11327238

[10] Wang XZ, Zhang SJ, Chen YX, Chen ZX, Huang YH, Zhang LJ. Effects of platelet-derived growth factor and interleukin-10 on Fas/Fas-ligand and Bcl-2/Bax mRNA expression in rat hepatic stellate cells in vitro. World journal of gastroenterology: WJG. 2004;10(18):2706–10. . 15309723 10.3748/wjg.v10.i18.2706 PMC4572197

[11] Yoshioka T, Okada T, Maeda Y, Ikeda U, Shimpo M, Nomoto T, et al Adeno-associated virus vector-mediated interleukin-10 gene transfer inhibits atherosclerosis in apolipoprotein E-deficient mice. Gene therapy. 2004;11(24):1772–9. 10.1038/sj.gt.3302348 . 15496963

[12] Pinderski Oslund LJ, Hedrick CC, Olvera T, Hagenbaugh A, Territo M, Berliner JA, et al Interleukin-10 blocks atherosclerotic events in vitro and in vivo. Arteriosclerosis, thrombosis, and vascular biology. 1999;19(12):2847–53. . 10591660 10.1161/01.atv.19.12.2847

[13] Von Der Thusen JH, Kuiper J, Fekkes ML, De Vos P, Van Berkel TJ, Biessen EA. Attenuation of atherogenesis by systemic and local adenovirus-mediated gene transfer of interleukin-10 in LDLr-/- mice. FASEB journal: official publication of the Federation of American Societies for Experimental Biology. 2001;15(14):2730–2. 10.1096/fj.01-0483fje . 11687507

[14] Han X, Kitamoto S, Wang H, Boisvert WA. Interleukin-10 overexpression in macrophages suppresses atherosclerosis in hyperlipidemic mice. FASEB journal: official publication of the Federation of American Societies for Experimental Biology. 2010;24(8):2869–80. 10.1096/fj.09-148155 20354139 PMC2909283

[15] Kimball AB, Kawamura T, Tejura K, Boss C, Hancox AR, Vogel JC, et al Clinical and immunologic assessment of patients with psoriasis in a randomized, double-blind, placebo-controlled trial using recombinant human interleukin 10. Archives of dermatology. 2002;138(10):1341–6. . 12374540 10.1001/archderm.138.10.1341

[16] Halvorsen B, Waehre T, Scholz H, Clausen OP, von der Thusen JH, Muller F, et al Interleukin-10 enhances the oxidized LDL-induced foam cell formation of macrophages by antiapoptotic mechanisms. Journal of lipid research. 2005;46(2):211–9. 10.1194/jlr.M400324-JLR200 . 15547296

[17] Rubic T, Lorenz RL. Downregulated CD36 and oxLDL uptake and stimulated ABCA1/G1 and cholesterol efflux as anti-atherosclerotic mechanisms of interleukin-10. Cardiovascular research. 2006;69(2):527–35. 10.1016/j.cardiores.2005.10.018 . 16336952

[18] Han X, Kitamoto S, Lian Q, Boisvert WA. Interleukin-10 facilitates both cholesterol uptake and efflux in macrophages. The Journal of biological chemistry. 2009;284(47):32950–8. 10.1074/jbc.M109.040899 19776020 PMC2781710

[19] Ross R, Glomset JA. Atherosclerosis and the arterial smooth muscle cell: Proliferation of smooth muscle is a key event in the genesis of the lesions of atherosclerosis. Science. 1973;180(4093):1332–9. . 4350926 10.1126/science.180.4093.1332

[20] Miller GJ, Miller NE. Plasma-high-density-lipoprotein concentration and development of ischaemic heart-disease. Lancet. 1975;1(7897):16–9. . 46338 10.1016/s0140-6736(75)92376-4

[21] Lewis GF, Rader DJ. New insights into the regulation of HDL metabolism and reverse cholesterol transport. Circulation research. 2005;96(12):1221–32. 10.1161/01.RES.0000170946.56981.5c . 15976321

[22] Wang X, Collins HL, Ranalletta M, Fuki IV, Billheimer JT, Rothblat GH, et al Macrophage ABCA1 and ABCG1, but not SR-BI, promote macrophage reverse cholesterol transport in vivo. The Journal of clinical investigation. 2007;117(8):2216–24. 10.1172/JCI32057 17657311 PMC1924499

[23] Ginsberg H, Grabowski GA, Gibson JC, Fagerstrom R, Goldblatt J, Gilbert HS, et al Reduced plasma concentrations of total, low density lipoprotein and high density lipoprotein cholesterol in patients with Gaucher type I disease. Clin Genet. 1984;26(2):109–16. . 6432380 10.1111/j.1399-0004.1984.tb00799.x

[24] de Fost M, Langeveld M, Franssen R, Hutten BA, Groener JE, de Groot E, et al Low HDL cholesterol levels in type I Gaucher disease do not lead to an increased risk of cardiovascular disease. Atherosclerosis. 2009;204(1):267–72. 10.1016/j.atherosclerosis.2008.08.027 . 18842264

[25] Reverter JC, Sierra J, Marti-Tutusaus JM, Montserrat E, Granena A, Rozman C. Hypocholesterolemia in acute myelogenous leukemia. Eur J Haematol. 1988;41(4):317–20. . 3197819 10.1111/j.1600-0609.1988.tb00203.x

[26] Budd D, Ginsberg H. Hypocholesterolemia and acute myelogenous leukemia. Association between disease activity and plasma low-density lipoprotein cholesterol concentrations. Cancer. 1986;58(6):1361–5. . 3461875 10.1002/1097-0142(19860915)58:6<1361::aid-cncr2820580630>3.0.co;2-s

[27] Tatidis L, Vitols S, Gruber A, Paul C, Axelson M. Cholesterol catabolism in patients with acute myelogenous leukemia and hypocholesterolemia: suppressed levels of a circulating marker for bile acid synthesis. Cancer Lett. 2001;170(2):169–75. . 11463495 10.1016/s0304-3835(01)00592-4

[28] Shiomi A, Usui T. Pivotal roles of GM-CSF in autoimmunity and inflammation. Mediators Inflamm. 2015;2015:568543 10.1155/2015/568543 25838639 PMC4370199

[29] Nimer SD, Champlin RE, Golde DW. Serum cholesterol-lowering activity of granulocyte-macrophage colony-stimulating factor. JAMA. 1988;260(22):3297–300. . 3054191

[30] Asadullah K, Docke WD, Ebeling M, Friedrich M, Belbe G, Audring H, et al Interleukin 10 treatment of psoriasis: clinical results of a phase 2 trial. Archives of dermatology. 1999;135(2):187–92. . 10052405 10.1001/archderm.135.2.187

[31] Reich K, Garbe C, Blaschke V, Maurer C, Middel P, Westphal G, et al Response of psoriasis to interleukin-10 is associated with suppression of cutaneous type 1 inflammation, downregulation of the epidermal interleukin-8/CXCR2 pathway and normalization of keratinocyte maturation. J Invest Dermatol. 2001;116(2):319–29. 10.1046/j.1523-1747.2001.01248.x . 11180010

[32] Tilg H, van Montfrans C, van den Ende A, Kaser A, van Deventer SJ, Schreiber S, et al Treatment of Crohn's disease with recombinant human interleukin 10 induces the proinflammatory cytokine interferon gamma. Gut. 2002;50(2):191–5. 11788558 10.1136/gut.50.2.191 PMC1773093

[33] Fedorak RN, Gangl A, Elson CO, Rutgeerts P, Schreiber S, Wild G, et al Recombinant human interleukin 10 in the treatment of patients with mild to moderately active Crohn's disease. The Interleukin 10 Inflammatory Bowel Disease Cooperative Study Group. Gastroenterology. 2000;119(6):1473–82. . 11113068 10.1053/gast.2000.20229

[34] Schreiber S, Fedorak RN, Nielsen OH, Wild G, Williams CN, Nikolaus S, et al Safety and efficacy of recombinant human interleukin 10 in chronic active Crohn's disease. Crohn's Disease IL-10 Cooperative Study Group. Gastroenterology. 2000;119(6):1461–72. . 11113067 10.1053/gast.2000.20196

[35] Herbert DR, Orekov T, Perkins C, Finkelman FD. IL-10 and TGF-beta redundantly protect against severe liver injury and mortality during acute schistosomiasis. Journal of immunology. 2008;181(10):7214–20. 18981143 10.4049/jimmunol.181.10.7214 PMC2921214

[36] Santucci L, Fiorucci S, Chiorean M, Brunori PM, Di Matteo FM, Sidoni A, et al Interleukin 10 reduces lethality and hepatic injury induced by lipopolysaccharide in galactosamine-sensitized mice. Gastroenterology. 1996;111(3):736–44. . 8780580 10.1053/gast.1996.v111.pm8780580

[37] Huang W, Metlakunta A, Dedousis N, Zhang P, Sipula I, Dube JJ, et al Depletion of liver Kupffer cells prevents the development of diet-induced hepatic steatosis and insulin resistance. Diabetes. 2010;59(2):347–57. 10.2337/db09-0016 19934001 PMC2809951

[38] Kolios G, Valatas V, Kouroumalis E. Role of Kupffer cells in the pathogenesis of liver disease. World journal of gastroenterology: WJG. 2006;12(46):7413–20. . 17167827 10.3748/wjg.v12.i46.7413 PMC4087584

[39] Klein I, Cornejo JC, Polakos NK, John B, Wuensch SA, Topham DJ, et al Kupffer cell heterogeneity: functional properties of bone marrow derived and sessile hepatic macrophages. Blood. 2007;110(12):4077–85. 10.1182/blood-2007-02-073841 17690256 PMC2190614

[40] Harkes L, Van Berkel JC. Quantitative role of parenchymal and non-parenchymal liver cells in the uptake of [14C]sucrose-labelled low-density lipoprotein in vivo. The Biochemical journal. 1984;224(1):21–7. 6508758 10.1042/bj2240021 PMC1144393

[41] Kamps JA, Kruijt JK, Kuiper J, Van Berkel TJ. Uptake and degradation of human low-density lipoprotein by human liver parenchymal and Kupffer cells in culture. The Biochemical journal. 1991;276 (Pt 1):135–40. 1903931 10.1042/bj2760135 PMC1151155

[42] Ichimura M, Kawase M, Masuzumi M, Sakaki M, Nagata Y, Tanaka K, et al High-fat and high-cholesterol diet rapidly induces non-alcoholic steatohepatitis with advanced fibrosis in Sprague-Dawley rats. Hepatol Res. 2015;45(4):458–69. 10.1111/hepr.12358 . 24827559

[43] Van Rooijen N, Sanders A. Liposome mediated depletion of macrophages: mechanism of action, preparation of liposomes and applications. J Immunol Methods. 1994;174(1–2):83–93. . 8083541 10.1016/0022-1759(94)90012-4

[44] Altmann SW, Davis HR Jr., Zhu LJ, Yao X, Hoos LM, Tetzloff G, et al Niemann-Pick C1 Like 1 protein is critical for intestinal cholesterol absorption. Science. 2004;303(5661):1201–4. 10.1126/science.1093131 . 14976318

[45] Davis HR Jr., Zhu LJ, Hoos LM, Tetzloff G, Maguire M, Liu J, et al Niemann-Pick C1 Like 1 (NPC1L1) is the intestinal phytosterol and cholesterol transporter and a key modulator of whole-body cholesterol homeostasis. The Journal of biological chemistry. 2004;279(32):33586–92. 10.1074/jbc.M405817200 . 15173162

[46] Istvan ES, Deisenhofer J. Structural mechanism for statin inhibition of HMG-CoA reductase. Science. 2001;292(5519):1160–4. 10.1126/science.1059344 . 11349148

[47] Peterson AS, Fong LG, Young SG. PCSK9 function and physiology. Journal of lipid research. 2008;49(7):1595–9. 18663786 10.1194/jlr.CX00001-JLR200 PMC3837453

[48] Lambert G, Sjouke B, Choque B, Kastelein JJ, Hovingh GK. The PCSK9 decade. Journal of lipid research. 2012;53(12):2515–24. 10.1194/jlr.R026658 22811413 PMC3494258

[49] Bell DA, Hooper AJ, Burnett JR. Mipomersen, an antisense apolipoprotein B synthesis inhibitor. Expert opinion on investigational drugs. 2011;20(2):265–72. 10.1517/13543784.2011.547471 . 21210756

[50] Thomas GS, Cromwell WC, Ali S, Chin W, Flaim JD, Davidson M. Mipomersen, an apolipoprotein B synthesis inhibitor, reduces atherogenic lipoproteins in patients with severe hypercholesterolemia at high cardiovascular risk: a randomized, double-blind, placebo-controlled trial. Journal of the American College of Cardiology. 2013;62(23):2178–84. 10.1016/j.jacc.2013.07.081 . 24013058

[51] Wetterau JR, Gregg RE, Harrity TW, Arbeeny C, Cap M, Connolly F, et al An MTP inhibitor that normalizes atherogenic lipoprotein levels in WHHL rabbits. Science. 1998;282(5389):751–4. . 9784135 10.1126/science.282.5389.751

[52] Allister EM, Borradaile NM, Edwards JY, Huff MW. Inhibition of microsomal triglyceride transfer protein expression and apolipoprotein B100 secretion by the citrus flavonoid naringenin and by insulin involves activation of the mitogen-activated protein kinase pathway in hepatocytes. Diabetes. 2005;54(6):1676–83. . 15919788 10.2337/diabetes.54.6.1676

[53] Cuchel M, Meagher EA, du Toit Theron H, Blom DJ, Marais AD, Hegele RA, et al Efficacy and safety of a microsomal triglyceride transfer protein inhibitor in patients with homozygous familial hypercholesterolaemia: a single-arm, open-label, phase 3 study. Lancet. 2013;381(9860):40–6. 10.1016/S0140-6736(12)61731-0 . 23122768 PMC4587657

[54] Martel C, Li W, Fulp B, Platt AM, Gautier EL, Westerterp M, et al Lymphatic vasculature mediates macrophage reverse cholesterol transport in mice. The Journal of clinical investigation. 2013;123(4):1571–9. 10.1172/JCI63685 23524964 PMC3613904

[55] Martel C, Randolph GJ. Atherosclerosis and transit of HDL through the lymphatic vasculature. Current atherosclerosis reports. 2013;15(9):354 10.1007/s11883-013-0354-4 23912686 PMC3799774

[56] Moore KJ, Sheedy FJ, Fisher EA. Macrophages in atherosclerosis: a dynamic balance. Nature reviews Immunology. 2013;13(10):709–21. 10.1038/nri3520 . 23995626 PMC4357520

[57] Bilzer M, Roggel F, Gerbes AL. Role of Kupffer cells in host defense and liver disease. Liver international: official journal of the International Association for the Study of the Liver. 2006;26(10):1175–86. 10.1111/j.1478-3231.2006.01342.x . 17105582

[58] Steib CJ. Kupffer cell activation and portal hypertension. Gut. 2011;60(10):1307–8. 10.1136/gut.2011.242560 . 21708827

[59] Harkes L, Van Berkel TJ. In vivo characteristics of a specific recognition site for LDL on non-parenchymal rat liver cells which differs from the 17 alpha-ethinyl estradiol-induced LDL receptor on parenchymal liver cells. Biochimica et biophysica acta. 1984;794(2):340–7. . 6329312 10.1016/0005-2760(84)90165-6

[60] Nenseter MS, Gudmundsen O, Roos N, Maelandsmo G, Drevon CA, Berg T. Role of liver endothelial and Kupffer cells in clearing low density lipoprotein from blood in hypercholesterolemic rabbits. Journal of lipid research. 1992;33(6):867–77. . 1324967

[61] Liu K, Czaja MJ. Regulation of lipid stores and metabolism by lipophagy. Cell Death Differ. 2013;20(1):3–11. 10.1038/cdd.2012.63 22595754 PMC3524634

[62] Tosello-Trampont AC, Landes SG, Nguyen V, Novobrantseva TI, Hahn YS. Kuppfer cells trigger nonalcoholic steatohepatitis development in diet-induced mouse model through tumor necrosis factor-alpha production. The Journal of biological chemistry. 2012;287(48):40161–72. 10.1074/jbc.M112.417014 23066023 PMC3504730

[63] Moore KW, de Waal Malefyt R, Coffman RL, O'Garra A. Interleukin-10 and the interleukin-10 receptor. Annual review of immunology. 2001;19:683–765. 10.1146/annurev.immunol.19.1.683 . 11244051

[64] Zhang LJ, Zheng WD, Chen YX, Huang YH, Chen ZX, Zhang SJ, et al Antifibrotic effects of interleukin-10 on experimental hepatic fibrosis. Hepato-gastroenterology. 2007;54(79):2092–8. . 18251166

[65] Nelson DR, Tu Z, Soldevila-Pico C, Abdelmalek M, Zhu H, Xu YL, et al Long-term interleukin 10 therapy in chronic hepatitis C patients has a proviral and anti-inflammatory effect. Hepatology. 2003;38(4):859–68. 10.1053/jhep.2003.50427 . 14512873

