# Supplementary figures and images for: PEGylated IL-10 Activates Kupffer Cells to Control Hypercholesterolemia
Source: PLoS One. 2016 Jun 14;11(6):e0156229. doi: 10.1371/journal.pone.0156229 (PMC4907428; doi:10.1371/journal.pone.0156229)

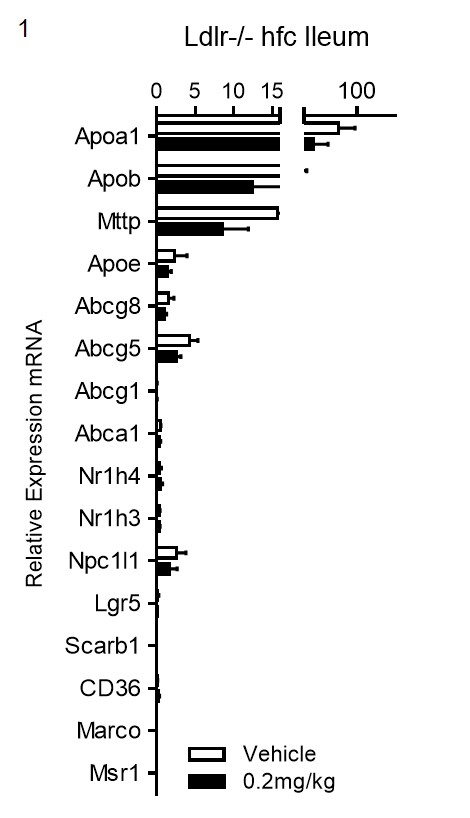

Supplement: S1 Fig — ileum mRNA expression profiling of Ldlr-/- fed high fat diet for 2 weeks and treated s.c. daily with 0.2 mg/kg PEG-rMuIL-10 for 2 weeks. (JPG) [file pone.0156229.s001.jpg]

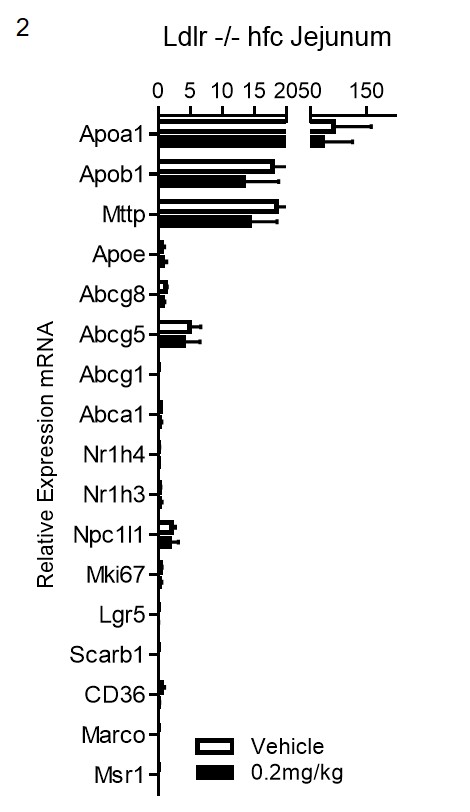

Supplement: S2 Fig — jejunum mRNA expression profiling of Ldlr-/- fed high fat diet for 2 weeks and treated s.c. daily with 0.2 mg/kg PEG-rMuIL-10 for 2 weeks. (JPG) [file pone.0156229.s002.jpg]

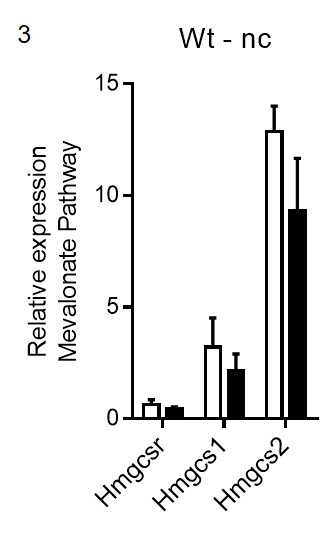

Supplement: S3 Fig — S3–S6 Figs, Expression of Mevalonate Pathway genes: 3-hydroxy-3-methyl-glutaryl-CoA reductase (Hmgcr), 3-hydroxyl-3-methyl-glutaryl-CoA Synthase 1 (Hmgcs1) and 3-hydroxyl-3-methyl-glutaryl-CoA Synthase 2 (Hmgcs2) were assessed by qPCR at the end of treatment. S3 Fig, wt mice on nc, were treated for 1–2 weeks with vehicle or 0.2 mg/kg s.c. qd PEG-rMuIL-10. (JPG) [file pone.0156229.s003.jpg]

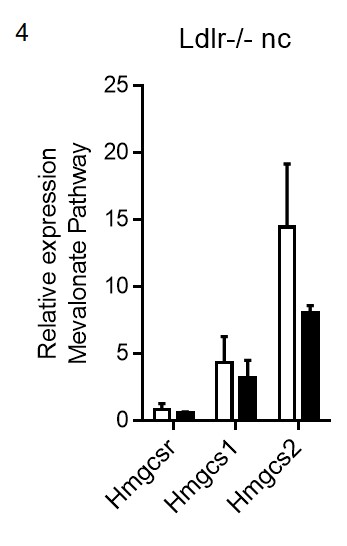

Supplement: S4 Fig — , Ldlr-/- mice on nc, were treated for 1–2 weeks with vehicle or 0.2 mg/kg s.c. qd PEG-rMuIL-10. (JPG) [file pone.0156229.s004.jpg]

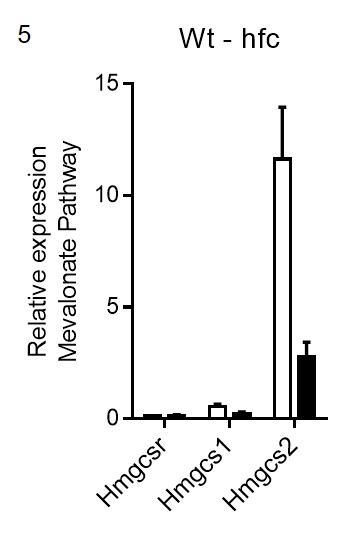

Supplement: S5 Fig — , wt mice mice on high fat chow (hfc) were fed for 2 weeks and treated for two weeks with vehicle or 0.2 mg/kg s.c. qd PEG-rMuIL-10. (JPG) [file pone.0156229.s005.jpg]

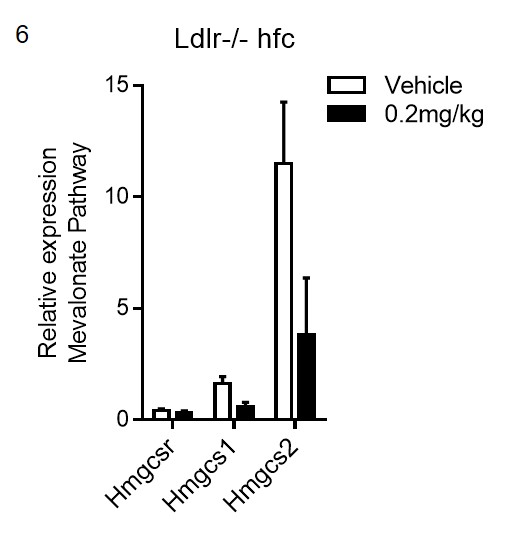

Supplement: S6 Fig — , Ldlr-/- mice on high fat chow (hfc) were fed for 2 weeks and treated for two weeks with vehicle or 0.2 mg/kg s.c. qd PEG-rMuIL-10. (JPG) [file pone.0156229.s006.jpg]

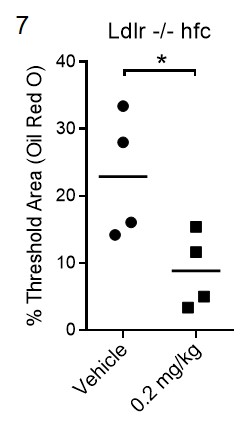

Supplement: S7 Fig — , Ldlr-/- mice were fed hfc for 2 weeks the dosed s.c. daily with 0.2 mg/kg PEG-rMuIL-10 for 2 weeks. 10–20 images per mouse were quantified with 4 mice per cohort randomly selected. The median percent threshold of signal was determined and plotted for each mouse. Wt or Ldlr-/- mice on nc were treated for 1–2 weeks with vehicle or 0.2 mg/kg s.c. qd PEG-rMuIL-10. Wt or Ldlr-/- mice on high fat chow (hfc) were fed for 4 weeks and treated with vehicle or 0.2 mg/kg s.c. qd PEG-rMuIL-10 during the last 2 weeks. Statistics assessed by Students T test where *p<0.05, **p<0.01, ***p<0.001. (JPG) [file pone.0156229.s007.jpg]

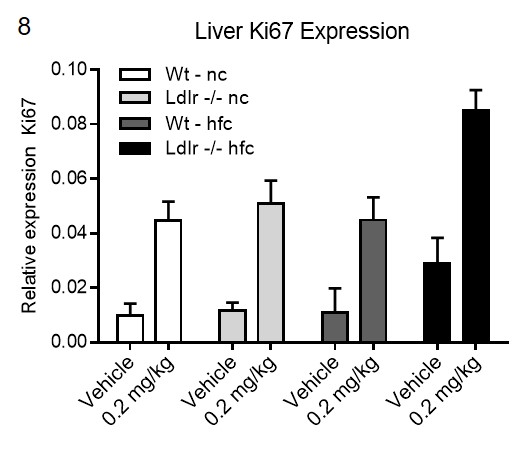

Supplement: S8 Fig — , hepatic expression analysis of Ki67 from 5–10 wt or LDLR-/- mice per group fed nc or hfc. (JPG) [file pone.0156229.s008.jpg]

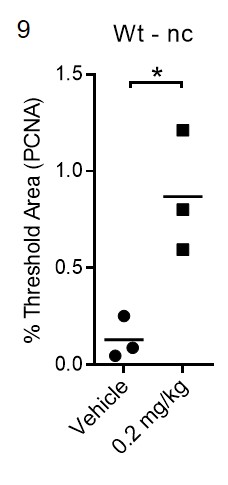

Supplement: S9 Fig — S9–S12 Figs, Liver PCNA IHC. 10–20 liver images per mouse were quantified with 2–3 mice per cohort randomly selected. The median percent threshold of signal was determined and plotted for each mouse. PCNA IHC image quantitation of wt mice on nc, S9 Fig, wt mice on nc. Statistics assessed by Students T test where *p<0.05, **p<0.01, ***p<0.001. (JPG) [file pone.0156229.s009.jpg]

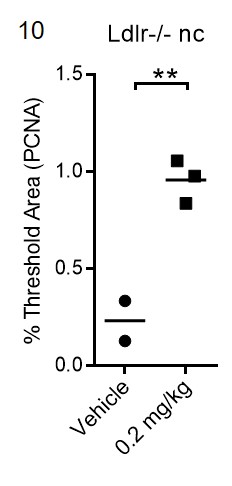

Supplement: S10 Fig — , Ldlr-/- mice fed nc. Statistics assessed by Students T test where *p<0.05, **p<0.01, ***p<0.001. (JPG) [file pone.0156229.s010.jpg]

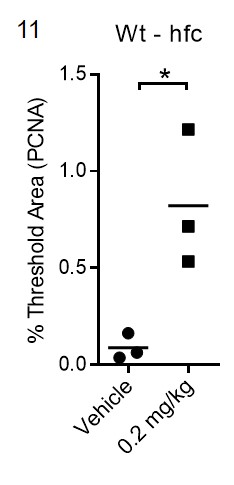

Supplement: S11 Fig — , wt mice on nc. Statistics assessed by Students T test where *p<0.05, **p<0.01, ***p<0.001. (JPG) [file pone.0156229.s011.jpg]

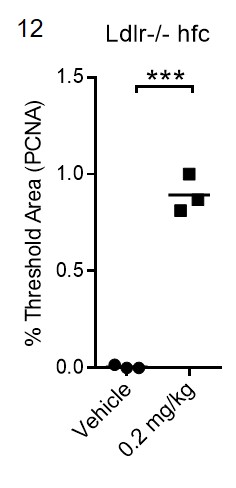

Supplement: S12 Fig — , Ldrl-/- on hfc. Statistics assessed by Students T test where *p<0.05, **p<0.01, ***p<0.001. (JPG) [file pone.0156229.s012.jpg]

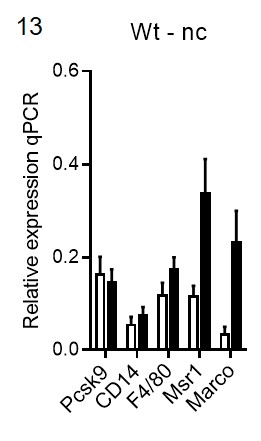

Supplement: S13 Fig — S13–S16 Figs, liver expression analysis of Pcsk9, CD14, F4/80, Msr1 and Marco genes from wt mice fed nc, S13 Fig, wt mice fed nc treated with vehicle or 0.2 mg/kg s.c. qd PEG-rMuIL-10. (JPG) [file pone.0156229.s013.jpg]

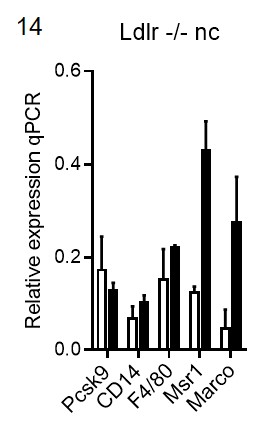

Supplement: S14 Fig — , Ldlr-/- mice fed nc treated with vehicle or 0.2 mg/kg s.c. qd PEG-rMuIL-10. (JPG) [file pone.0156229.s014.jpg]

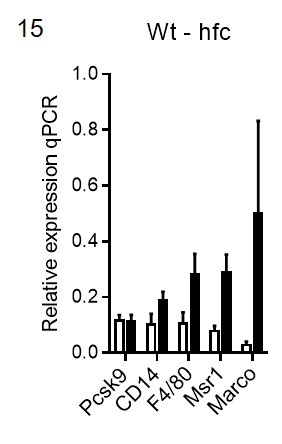

Supplement: S15 Fig — , wt mice fed hfc treated with vehicle or 0.2 mg/kg s.c. qd PEG-rMuIL-10. (JPG) [file pone.0156229.s015.jpg]

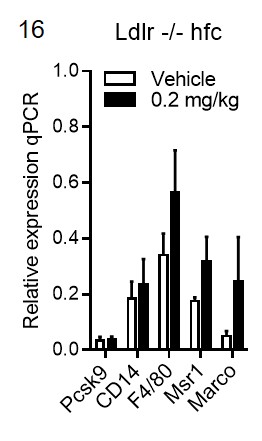

Supplement: S16 Fig — , Ldlr-/- mice fed hfc treated with vehicle or 0.2 mg/kg s.c. qd PEG-rMuIL-10. (JPG) [file pone.0156229.s016.jpg]

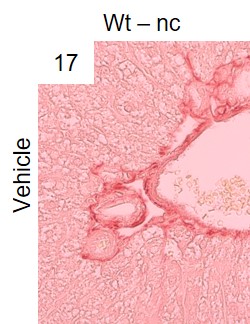

Supplement: S17 Fig — Liver Sirius Red IHC. S17–S20 Figs, representative periportal liver images of vehicle dosed wt and Ldlr-/- mice on nc and hfc. S21–S24 Figs, representative periportal images of 0.2 mg/kg s.c. qd PEG-rMuIL-10 dosed wt and LDLR-/- mice fed nc and hfc. S17 Fig, wt fed nc, vehicle treated for 1 week. (JPG) [file pone.0156229.s017.jpg]

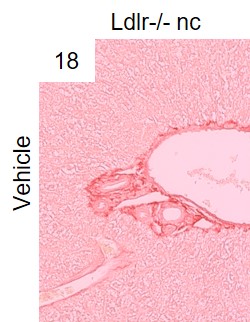

Supplement: S18 Fig — , Ldlr-/- fed nc, vehicle treated for 1 week. (JPG) [file pone.0156229.s018.jpg]

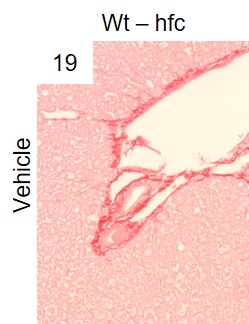

Supplement: S19 Fig — , wt on hfc, vehicle treated for 2 weeks. (JPG) [file pone.0156229.s019.jpg]

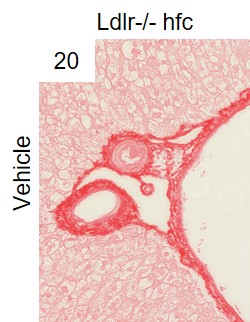

Supplement: S20 Fig — , Ldlr-/- fed hfc, vehicle treated for 2 weeks. (JPG) [file pone.0156229.s020.jpg]

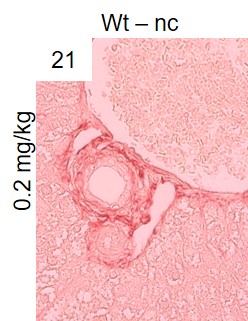

Supplement: S21 Fig — , wt fed nc, PEG-rMuIL-10 treated for 1 week. (JPG) [file pone.0156229.s021.jpg]

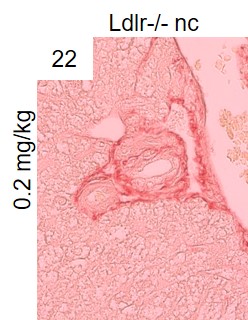

Supplement: S22 Fig — , Ldlr-/- fed nc, PEG-rMuIL-10 treated for 1 week. (JPG) [file pone.0156229.s022.jpg]

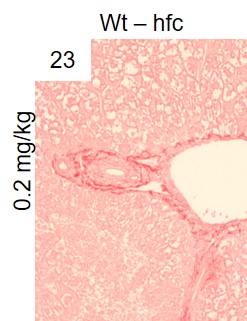

Supplement: S23 Fig — , wt fed hfc for 4 weeks, treated with PEG-rMuIL-10 for 2 weeks. (JPG) [file pone.0156229.s023.jpg]

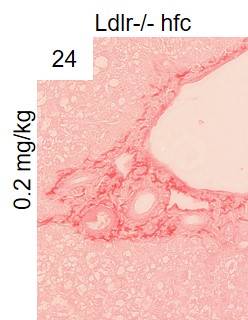

Supplement: S24 Fig — , Ldlr-/- fed hfc for 4 weeks, treated with PEG-rMuIL-10 for 2 weeks. (JPG) [file pone.0156229.s024.jpg]

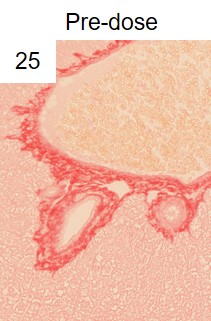

Supplement: S25 Fig — S25Fig, Ldlr-/- fed hfc for 7 weeks. (JPG) [file pone.0156229.s025.jpg]

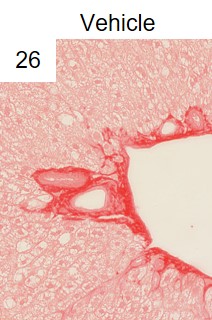

Supplement: S26 Fig — , Ldlr-/- from S25 Fig, dosed with vehicle for 3 weeks while remaining on hfc. (JPG) [file pone.0156229.s026.jpg]

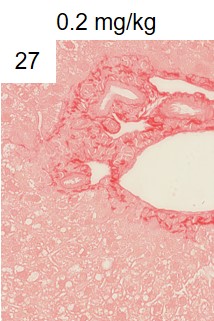

Supplement: S27 Fig — , Ldlr-/- from S25 Fig, dosed with 0.2mg/kg for 3 weeks with 0.2mg/kg PEG-rMuIL-10 s.c. qd while remaining on hfc. (JPG) [file pone.0156229.s027.jpg]

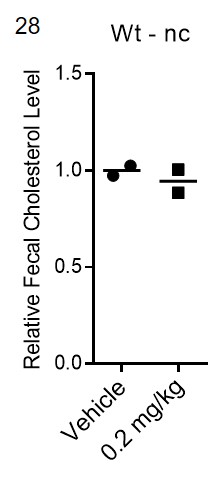

Supplement: S28 Fig — Mice treated as in S28–S31 Figs. Fecal bile acids and cholesterol measurements represent the total fecal matter from 2 weeks of dosing from two separate cages housing 4–5 mice per cage. To ensure sufficient material to quantify after drying and extraction, the total fecal material from each cage had to be pooled. S28 Fig fecal cholesterol of wt mice fed nc. (JPG) [file pone.0156229.s028.jpg]

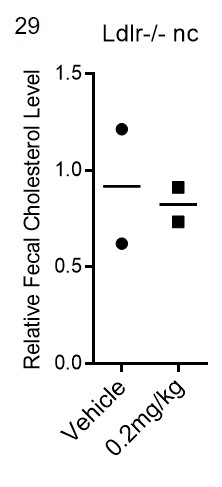

Supplement: S29 Fig — , fecal cholesterol of Ldlr-/- mice fed nc. (JPG) [file pone.0156229.s029.jpg]

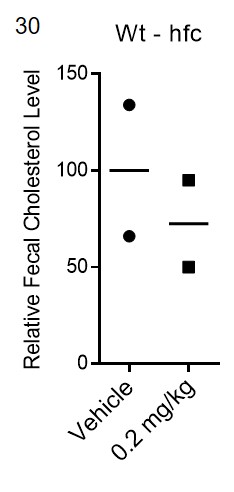

Supplement: S30 Fig — , fecal cholesterol of wt and mice fed high fat chow (hfc). (JPG) [file pone.0156229.s030.jpg]

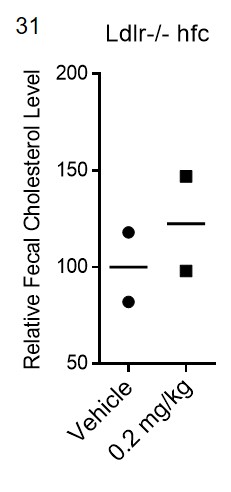

Supplement: S31 Fig — , fecal cholesterol of Ldlr-/- mice fed high fat chow (hfc). (JPG) [file pone.0156229.s031.jpg]

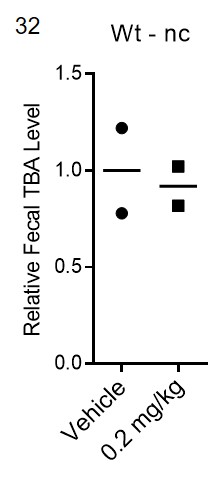

Supplement: S32 Fig — , fecal bile acids of wt mice fed nc. (JPG) [file pone.0156229.s032.jpg]

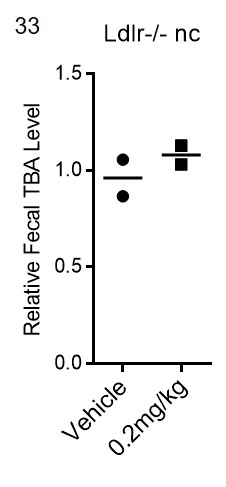

Supplement: S33 Fig — , fecal bile acids of wt and Ldlr-/- mice fed nc. (JPG) [file pone.0156229.s033.jpg]

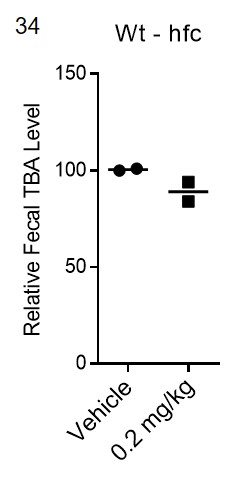

Supplement: S34 Fig — , fecal bile acids of wt mice fed hfc. (JPG) [file pone.0156229.s034.jpg]

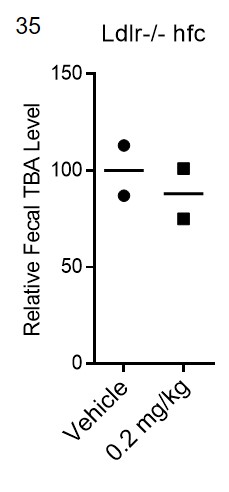

Supplement: S35 Fig — , fecal bile acids of Ldlr-/- mice fed hfc. (JPG) [file pone.0156229.s035.jpg]

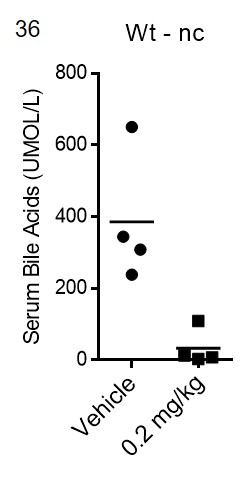

Supplement: S36 Fig — , serum bile acids in wt mice fed nc. (JPG) [file pone.0156229.s036.jpg]

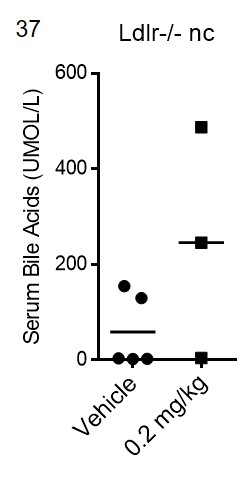

Supplement: S37 Fig — , serum bile acids in Ldlr-/- mice fed nc. (JPG) [file pone.0156229.s037.jpg]

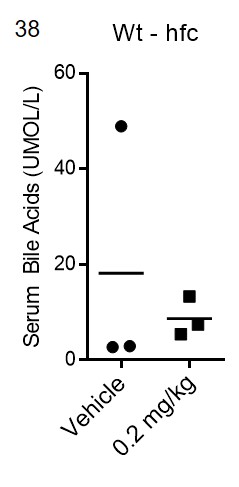

Supplement: S38 Fig — , serum bile acids in wt mice fed hfc. (JPG) [file pone.0156229.s038.jpg]

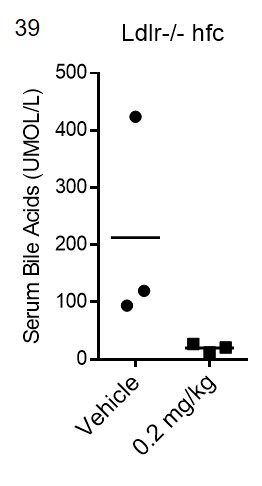

Supplement: S39 Fig — , serum bile acids in Ldlr-/- mice fed hfc. (JPG) [file pone.0156229.s039.jpg]

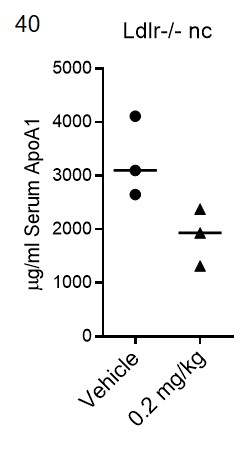

Supplement: S40 Fig — serum ApoA1 in Ldlr-/- mice fed nc. (JPG) [file pone.0156229.s040.jpg]

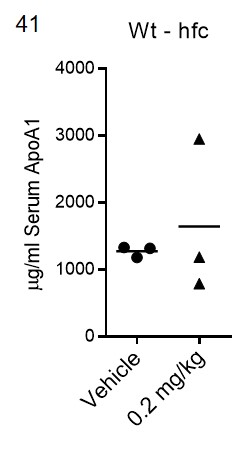

Supplement: S41 Fig — , serum ApoA1 in wt mice fed hfc. (JPG) [file pone.0156229.s041.jpg]

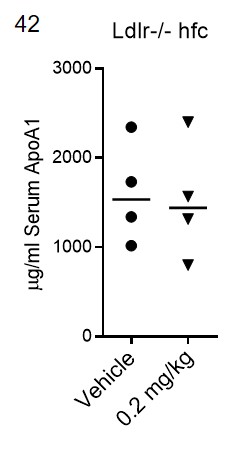

Supplement: S42 Fig — , serum ApoA1 in Ldlr-/-mice fed hfc. (JPG) [file pone.0156229.s042.jpg]

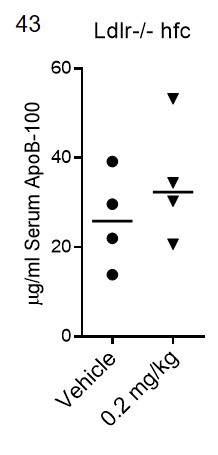

Supplement: S43 Fig — , serum ApoB-100 in Ldlr-/- mice fed hfc. (JPG) [file pone.0156229.s043.jpg]

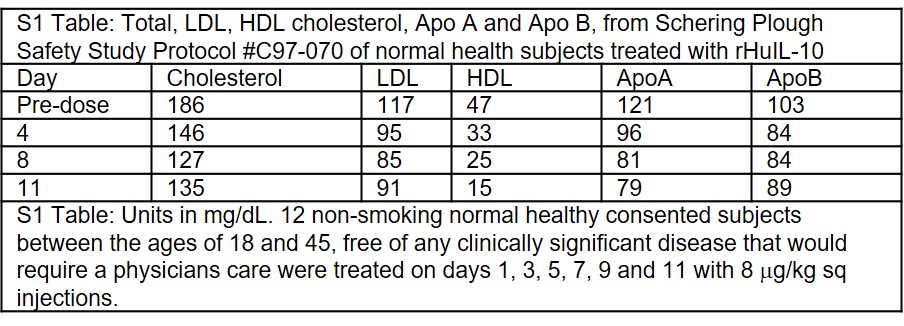

Supplement: S1 Table — , Serum chemistry data from Schering Plough safety study in health volunteers. Cholesterol, LDL, HDL, AopA and ApoB levels from 12 heathy donors treated subcutaneous (s.c.), every other day with 8 μg/kg rHuIL-10 for 11 days. Serum chemistry was analyzed pre-dose and on day 12. (JPG) [file pone.0156229.s044.jpg]

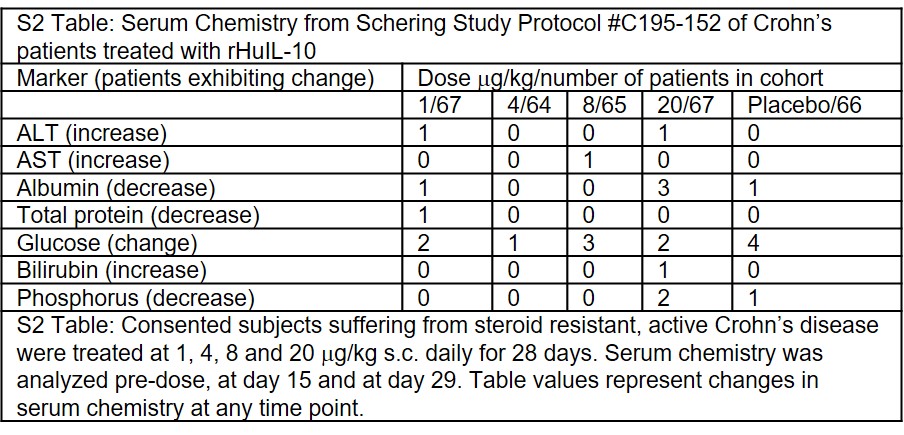

Supplement: S2 Table — , Serum chemistry data from Schering Plough Crohn’s study. 263 total patients with active steroid refractory Crohn’s disease were treated s.c., daily (qd), for 28 days with 1, 4, 8, 20 μg/kg rHuIL-10 or placebo. S2 Table lists serum chemistry markers and the number of patients who exhibited changes in these markers at any time during the study. (JPG) [file pone.0156229.s045.jpg]

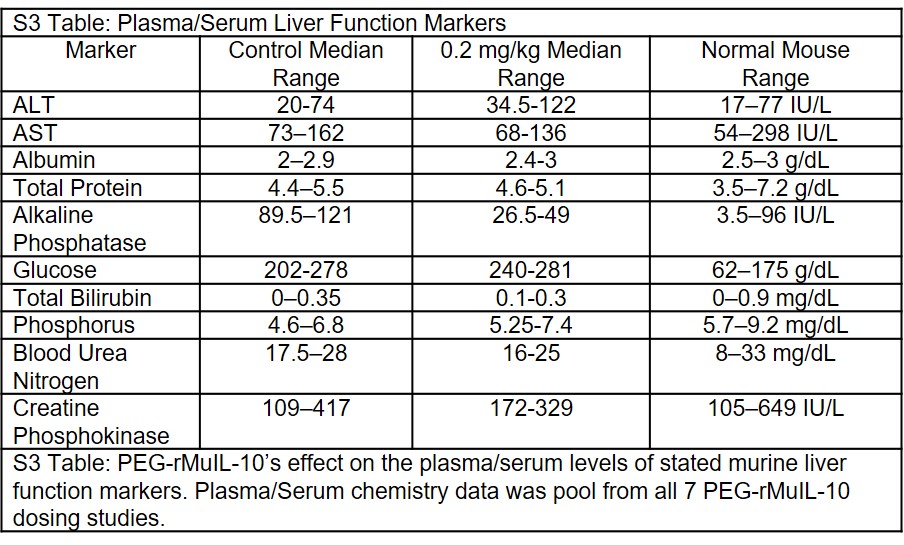

Supplement: S3 Table — , Serum chemistry data was pooled from all 7 PEG-rMuIL-10 dosing studies. S3 Table illustrates the plasma/serum levels after dosing with 0.2 mg/kg PEG-rMuIL-10 for listed liver function markers. (JPG) [file pone.0156229.s046.jpg]
